# Talin2 and KANK2 functionally interact to regulate microtubule dynamics, paclitaxel sensitivity and cell migration in the MDA-MB-435S melanoma cell line

**DOI:** 10.1186/s11658-023-00473-6

**Published:** 2023-07-17

**Authors:** Marija Lončarić, Nikolina Stojanović, Anja Rac-Justament, Kaatje Coopmans, Dragomira Majhen, Jonathan D. Humphries, Martin J. Humphries, Andreja Ambriović-Ristov

**Affiliations:** 1 Laboratory for Cell Biology and Signalling, Division of Molecular Biology, Ruđer Bošković Institute, Zagreb, Croatia; 2 Department of Life Science, Manchester Metropolitan University, Manchester, United Kingdom; 3 Wellcome Centre for Cell-Matrix Research, Faculty of Biology, Medicine and Health, University of Manchester, Manchester, United Kingdom

**Keywords:** Focal adhesion, Talin1, Talin2, KANK1, KANK2, Cortical microtubule stabilizing complex

## Abstract

**Background:**

Focal adhesions (FAs) are integrin-containing, multi-protein structures that link intracellular actin to the extracellular matrix and trigger multiple signaling pathways that control cell proliferation, differentiation, survival and motility. Microtubules (MTs) are stabilized in the vicinity of FAs through interaction with the components of the cortical microtubule stabilizing complex (CMSC). KANK (KN motif and ankyrin repeat domains) family proteins within the CMSC, KANK1 or KANK2, bind talin within FAs and thus mediate actin-MT crosstalk. We previously identified in MDA-MB-435S cells, which preferentially use integrin αVβ5 for adhesion, KANK2 as a key molecule enabling the actin-MT crosstalk. KANK2 knockdown also resulted in increased sensitivity to MT poisons, paclitaxel (PTX) and vincristine and reduced migration. Here, we aimed to analyze whether KANK1 has a similar role and to distinguish which talin isoform binds KANK2.

**Methods:**

The cell model consisted of human melanoma cell line MDA-MB-435S and stably transfected clone with decreased expression of integrin αV (3αV). For transient knockdown of talin1, talin2, KANK1 or KANK2 we used gene-specific siRNAs transfection. Using previously standardized protocol we isolated integrin adhesion complexes. SDS-PAGE and Western blot was used for protein expression analysis. The immunofluorescence analysis and live cell imaging was done using confocal microscopy. Cell migration was analyzed with Transwell Cell Culture Inserts. Statistical analysis using GraphPad Software consisted of either one-way analysis of variance (ANOVA), unpaired Student’s *t*-test or two-way ANOVA analysis.

**Results:**

We show that KANK1 is not a part of the CMSC associated with integrin αVβ5 FAs and its knockdown did not affect the velocity of MT growth or cell sensitivity to PTX. The talin2 knockdown mimicked KANK2 knockdown i.e. led to the perturbation of actin-MT crosstalk, which is indicated by the increased velocity of MT growth and increased sensitivity to PTX and also reduced migration.

**Conclusion:**

We conclude that KANK2 functionally interacts with talin2 and that the mechanism of increased sensitivity to PTX involves changes in microtubule dynamics. These data elucidate a cell-type-specific role of talin2 and KANK2 isoforms and we propose that talin2 and KANK2 are therefore potential therapeutic targets for improved cancer therapy.

**Graphical Abstract:**

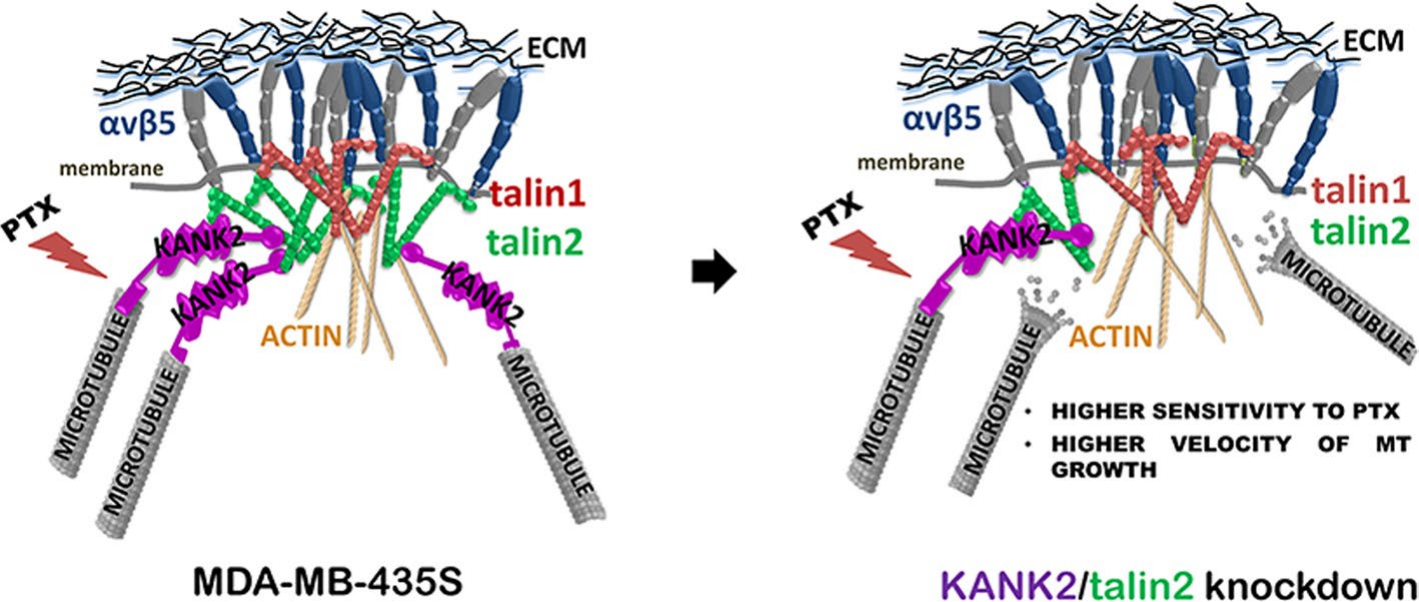

**Supplementary Information:**

The online version contains supplementary material available at 10.1186/s11658-023-00473-6.

## Background

Integrins are cell surface adhesion receptors composed of α and β subunits, forming 24 different αβ heterodimers that connect cells either to other cells or to the extracellular matrix (ECM) [1, 2]. Binding of ECM ligands results in the formation of several functionally and structurally different classes of integrin adhesion complexes (IACs). These include nascent adhesions, focal adhesions (FAs) and fibrillar adhesions, which serve as a link to the actin cytoskeleton [3], hemidesmosomes, which link to intermediate filaments [4], and reticular adhesions (RAs), which are not associated to the cytoskeleton [5]. IACs activate a range of signaling pathways, which in turn control different physiological processes such as cell survival, differentiation, proliferation, migration and metabolism [2].

Focal adhesions are the most extensively studied IACs involved in the conversion of extracellular chemical and mechanical cues into biochemical signals [6–9]. Microtubules (MTs) regulate both FA assembly [10, 11] and turnover during cell migration [12, 13] by interacting with proximally-located cortical microtubule stabilizing complexes (CMSCs) [14] or plasma membrane-associated platforms (PMAPs) [15]. These complexes contain liprins α and β [16], ELKS (proteins rich in amino acids E, L, K and S) [17], kinesin family member KIF21A [18], plus-end tracking protein EB1, CLASPs (CLIP-associating proteins) and LL5β [11, 16, 18, 19], as well as KN motif and ankyrin repeat domains (KANKs) family proteins [14, 20–22], all of which have been detected in biochemically-isolated IACs using mass spectrometry (MS)-based proteomics [23, 24]. KANKs, which bind directly to talins, are essential for connecting the CMSC to integrins [14, 20], but has not yet been established which isoforms contribute to the connection and whether their binding is cell type-specific.

Talins bind directly to the cytoplasmic domain of β integrin subunits via their FERM domain. The integrin-talin connection, supported further by binding of vinculin to talin, links integrins to actin [25]. Two isoforms, talin1 and talin2, are expressed by two separate genes but share a highly conserved structure composed of a head domain and 13 rod domains [26]. Knockout of talin1, but not talin2, is lethal in mice [27, 28]. KANKs bind to the R7 rod domain of talin, anchoring and stabilizing MTs in close proximity to FAs [14, 20]. The four KANK family isoforms (1–4) possess three distinct domains, a KANK N-terminal (KN) domain, a coiled-coil (CC) domain whose composition and number varies among the family members, and an ankyrin repeat domain (ANKRD), which consists of five ankyrin repeats [14, 20–22, 29]. The KN domain binds to talin, the CC domain to liprin-β1 and ANKRD to KIF21A [18, 30, 31].

The composition of IACs of different cell lines (termed the adhesome) has been catalogued following biochemical isolation or in situ followed by proteomic analysis [32]. Isolated IACs have primarily been generated from cells seeded for short periods of time on fibronectin [33, 34], but also from cells in long-term culture where the cells secrete their own ECM, which is then used for binding of available integrin heterodimers. The latter method has shown that cells predominantly use a single integrin, whether it is organized as FAs and/or RAs [5, 24], or as hemidesmosomes [35, 36]. The advantage of using cell models in which IACs are already explored and are composed of preferentially one integrin heterodimer facilitates the research of the role of individual molecules in a specific integrin-forming IACs.

Our previous study showed that the melanoma cell line MDA-MB-435S preferentially uses integrin αVβ5 for adhesion to uncoated surfaces and identified key components of its adhesome [24]. Following integrin αV knockdown we observed increased sensitivity to MT poisons, paclitaxel (PTX) and vincristine, and decreased migration. αVβ5 was responsible for both effects [37]. By comparing the composition of IACs in MDA-MB-435S cells to cells in which integrin αV was stably knocked down, we identified the components of the integrin αVβ5 adhesome. Proteins decreased after αV knockdown included talin1, talin2 and all the components of CMSC, including KANK2, whose knockdown in MDA-MB-435S cells mimicked the effect of integrin αV knockdown and resulted in increased sensitivity to MT poisons and decreased migration. We concluded that KANK2 is a key molecule linking integrin αVβ5 IACs to MTs, enabling the actin-MT crosstalk important for both sensitivity to MT poisons and cell migration [24]. However, according to the (MS)-based proteomic analysis, these cells also express KANK1 at a low level. Furthermore, it was not possible to deduce which talin isoform plays a role in binding KANK1 or KANK2 proteins, and what is the exact mechanism of altered sensitivity to PTX.

The aim of the present work was to distinguish which talin isoform binds KANK2 and whether KANK1 has a similar role to KANK2. In addition, our aim was to further elucidate the mechanism of increased sensitivity to PTX upon integrin depletion. We show that KANK1 does not colocalize with αVβ5 FAs. Moreover, KANK1 is not altered after stable integrin αV knockdown and its knockdown does not change the sensitivity to PTX. We show that KANK2 and talin2 functionally interact thus enabling the actin-MT crosstalk important for both sensitivity to MT poisons and cell migration. We also show that the mechanism by which this occurs involves changes in microtubule dynamics. Since talin2 or KANK2 knockdown mimicked the effect of integrin αV knockdown and resulted in increased sensitivity to PTX and reduced migration, talin2 and KANK2 are potential therapeutic targets for improved PTX therapy and reduced metastasis which might have a more uniform response than targeting integrins.

## Materials and methods

### Cell cultures and generation of stably transfected cells

The human melanoma cell line MDA-MB-435S (a spindle-shaped variant of the parental MDA-MB-435) was obtained from the American Type Culture Collection (ATCC). The isolation of the MDA-MB-435S-derived cell clone with decreased expression of integrin αV (3αV clone) was described previously [24]. MDA-MB-435S and 3αV cell populations with fluorescently labelled end-binding protein 3 (EB3) were generated by stable transfection of Dendra2-EB3-7 plasmid containing the photoconvertible fluorescent protein Dendra2 fused with EB3 (EB3-Dendra2) (Addgene plasmid #57715) using Lipofectamine 2000 (Thermo Fisher Scientific) and selection with 1.2 mg/mL geneticin (Sigma-Aldrich). Cells were grown in DMEM (Invitrogen) supplemented with 10% (v/v) FBS (Invitrogen) (DMEM-FBS) at 37 °C with 5% CO_2_ (v/v) in a humidified atmosphere.

All the experiments in our study were done with cells that were seeded on uncoated surfaces. The reason for this is that we published previously that MDA-MB-435S cells preferentially use integrin αVβ5 for adhesion and that, in such conditions these cells become more sensitive to paclitaxel upon integrin αV or KANK2 knockdown [24].

### Transient siRNA transfection

For transient siRNA transfection experiments, cells (4 × 10^5^) were seeded in 3.5 cm Petri dishes and transfected 24 h later, using Lipofectamine RNAiMax (13778150, Thermo Fisher Scientific), with 25 nM of control (Silencer™ Select Negative Control No. 1 siRNA, Ambion) or gene-specific siRNA for KANK1 (target sequence: CAGAGAAGGACATGCGGAT), KANK2 (target sequence: ATGTCAACGTGCAAGATGA), Talin1 (target sequence: TGAATGTCCTGTCAACTGCTG) or Talin2 (target sequence: TTTCGTTTTCATCTACTCCTT) [14], all purchased from Sigma. Successful knockdown was validated by SDS-PAGE and western blotting (WB) using specific antibodies and matched labelled secondary antibodies. The primary and secondary antibodies are listed in Additional file 9: Table S1.

### Isolation of IACs

Integrin adhesion complexes were isolated from cells cultivated for 48 h as previously described [24, 38]. Briefly, cells were washed with DMEM-HEPES and incubated with Wang and Richard’s reagent (DTBP, 6 mM, Thermo Fisher Scientific) for 10 min. DTBP was quenched with 0.03 M Tris–HCl (pH 8) and cells were lysed using modified RIPA buffer (50 mM Tris–HCl, pH 7.6; 150 mM NaCl; 5 mM disodium EDTA, pH 8; 1% (w/v) Triton X-100, 0.5% (w/v) SDS, 1% (w/v) sodium deoxycholate). Cell bodies were removed by high-pressure washing with tap water for 10 s and remaining adhesion complexes were collected by scraping into adhesion recovery solution (125 mM Tris–HCl, pH 6.8; 1% (w/v) SDS; 150 mM dithiothreitol). Samples containing isolated IACs were acetone-precipitated and further processed for WB analysis [33].

### SDS-PAGE and western blotting

Total cell lysates were obtained from 3.5 cm Petri dishes in 200 µL RIPA buffer supplemented with protease inhibitor cocktail (ThermoFisher). Samples for SDS-PAGE were collected by scraping. Samples containing an equal amount of protein were mixed in 6 × Laemmli loading buffer (375 mM Tris–HCl (pH 6.8), 30% (w/v) glycerol, 12% (w/v) SDS, 0.02% (w/v) bromophenol blue, 12% (v/v) 2-mercaptoethanol) to reach a final 1 × concentration, sonicated and heated for 5 min at 96 °C. Isolated IACs were prepared for SDS-PAGE by solubilization in 2 × Laemmli loading buffer and heating for 20 min at 70 °C while shaking (1000 rpm). All samples were loaded onto pre-casted gradient gel (4–15% Mini-PROTEAN TGX) (Bio-Rad), separated by SDS-PAGE and semi-dry transferred to nitrocellulose (Bio-Rad). The membrane was blocked in 5% (w/v) non-fat dry milk or 5% (w/v) bovine serum albumin (BSA, Carl Roth), and incubated overnight with the appropriate antibodies, followed by incubation with horseradish peroxidase coupled secondary antibody. The primary and secondary antibodies are listed in Additional file 9: Table S1. Detection was performed using chemiluminescence (PerkinElmer) and documented with Uvitec Alliance Q9 mini (BioSPX b.v.). Blots were quantified using ImageJ.

### Cell survival analysis

An MTT (3-((4,5-dimethylthiazol-2-yl)-2,5-diphenyltetrazolium bromide) (Millipore) assay was used to determine sensitivity of cells to PTX (Sigma-Aldrich). Briefly, 24 h after seeding in 96-well tissue culture plates (1–1.2 × 10^4^ cells/well) cells were treated with different concentrations of PTX. Seventy-two hours later, the absorbance of MTT-formazan product dissolved in dimethyl sulfoxide was measured with a microplate reader (Awareness Technology, Inc.) at 600 nm. Measured absorbance was proportional to the number of viable cells.

### Confocal microscopy and live cell imaging

For immunofluorescence analysis (IF), cells were plated on coverslips in a 24-well plate (3.5 × 10^4^ cells/well). After 48 h, cells were fixed using ice-cold methanol for 10 min or 2% (v/v) paraformaldehyde for 12 min followed by permeabilization with 0.1% (v/v) Triton X-100 for 2 min, and stained with protein specific primary antibodies for 1 h, followed by incubation with conjugated secondary antibodies for 1 h. The primary and secondary antibodies are listed in Additional file 9: Table S1. Actin stress fibers were stained with rhodamine phalloidin (Cell Signaling Technology). Cells were mounted with DAPI Fluoromount-G (SouthernBiotech) and fluorescence and respective IRM images were acquired using an inverted confocal microscope (Leica TCS SP8 X, Leica Microsystems) with the HC PL APOCS2 63 × /1.40 oil-immersion objective, zoom set at 2 × . Images were analyzed using LAS X software 3.1.1 (Leica Microsystems) and ImageJ. For actin stress fibers quantification, only those fibers that ended with FAs, marked by integrin β5 staining, were identified as stress fibers and quantified. For microtubule quantification, only microtubules within 5 µm from the cell edge (identified through IRM images) were quantified. All images were taken with the focus adjusted to the adhesion sites of cells at the upper surface of glass coverslip.

Time-lapse live cell microscopy of MDA-MB-435S-EB3 and 3αV-EB3 cells was performed 48 h upon seeding in a 4-chamber 35 mm Cellvis glass bottom dish (4.5 × 10^4^ cells/chamber). In experiments with transient knockdown or PTX treatment, cells were imaged 48 h after transfection or 24 h after treatment with PTX. Images were taken every 13 s for five min per cell using HC PL APO CS2 63 × /1.40 oil-immersion objective on the Leica TCS SP8 X microscope (Leica Microsystems), 3.5 × zoom. Obtained movies were analyzed with ImageJ software (Manual tracking plugin).

### Cell migration

Serum-starved (24 h) cells (8 × 10^4^) were placed in Transwell Cell Culture Inserts (pore size, 8 µm) (Corning) in DMEM containing 0.1% (w/v) BSA) and left to migrate toward 10% (v/v) FBS in DMEM as a chemoattractant. After 22 h, cells that remained on the upper side of the inserts were removed with cotton-tipped swabs. Inserts were fixed in 4% paraformaldehyde for 15 min followed by staining with 1% (w/v) crystal violet in PBS for 90 min. Cells on the underside of the inserts were photographed using Olympus BX51TF microscope (five images/sample). The number of cells was determined using ImageJ software (Multi-point tool).

### Cell proliferation assay

To determine cell proliferation upon knockdown of each talin and KANK, Click-iT assay was used according to the manufacturer’s instructions (Thermo Fisher Scientific, United States). Cells were seeded in a 6-well plate (3.5 × 10^5^ cells/well) and upon 24 h transfected with either control siRNA or specific siRNA. After 24 h, cells were trypsinized and seeded back in the same well to mimic steps during MTT assay. Next day, two hours before harvesting, modified thymidine analog EdU (5-ethynyl-2’-deoxyuridine, final concentration 10 µM) was added. Cells were collected, fixed with 4% (w/v) paraformaldehyde, permeabilized with saponin, stained with Alexa-Fluor 488 azide (in the presence of CuSO_4_) and analyzed by flow cytometry. Flow cytometry was performed using BD FACSCalibur (BD Biosciences, United States). Data were analyzed using FCS Express (De Novo Software, United States). To determine the proliferation rate, the frequencies of the proliferative (EdU +) cells were compared.

### Statistical analysis

GraphPad Prism version 9.0.0 (GraphPad Software) was used to analyze data. Data obtained from IF, WB, proliferation and migration assay were analyzed by one-way analysis of variance (ANOVA) with Dunnett’s multiple comparison or by unpaired Student’s *t*-test, and expressed as histograms depicting mean ± standard deviation (SD); violin plots or scatter plots with marked median; ns denotes not significant; * denotes *p* < 0.05; ** denotes *p* < 0.01; *** denotes *p* < 0.001; **** denotes *p* < 0.0001. Data from time-lapse live cell microscopy were analyzed by ordinary one-way ANOVA with Šídák’s multiple comparisons test, with a single pooled variance and shown as violin plots or scatter plots with marked median; ns denotes not significant; * denotes *p* < 0.05; ** denotes *p* < 0.01; *** denotes *p* < 0.001; **** denotes *p* < 0.0001 or by unpaired Student’s *t*-test, and expressed as mean ± SD; ns denotes not significant; * denotes *p* < 0.05; ** denotes *p* < 0.01; *** denotes *p* < 0.001; **** denotes *p* < 0.0001. Data obtained from MTT experiments were analyzed by two-way ANOVA with Šídák’s multiple comparisons test, with a single pooled variance and expressed as mean ± SD; ns denotes not significant; * denotes *p* < 0.05; ** denotes *p* < 0.01; *** denotes *p* < 0.001; **** denotes *p* < 0.0001.

## Results

### Talin isoform specific regulation of integrin αVβ5 and KANK localization

**Fig. 1:**
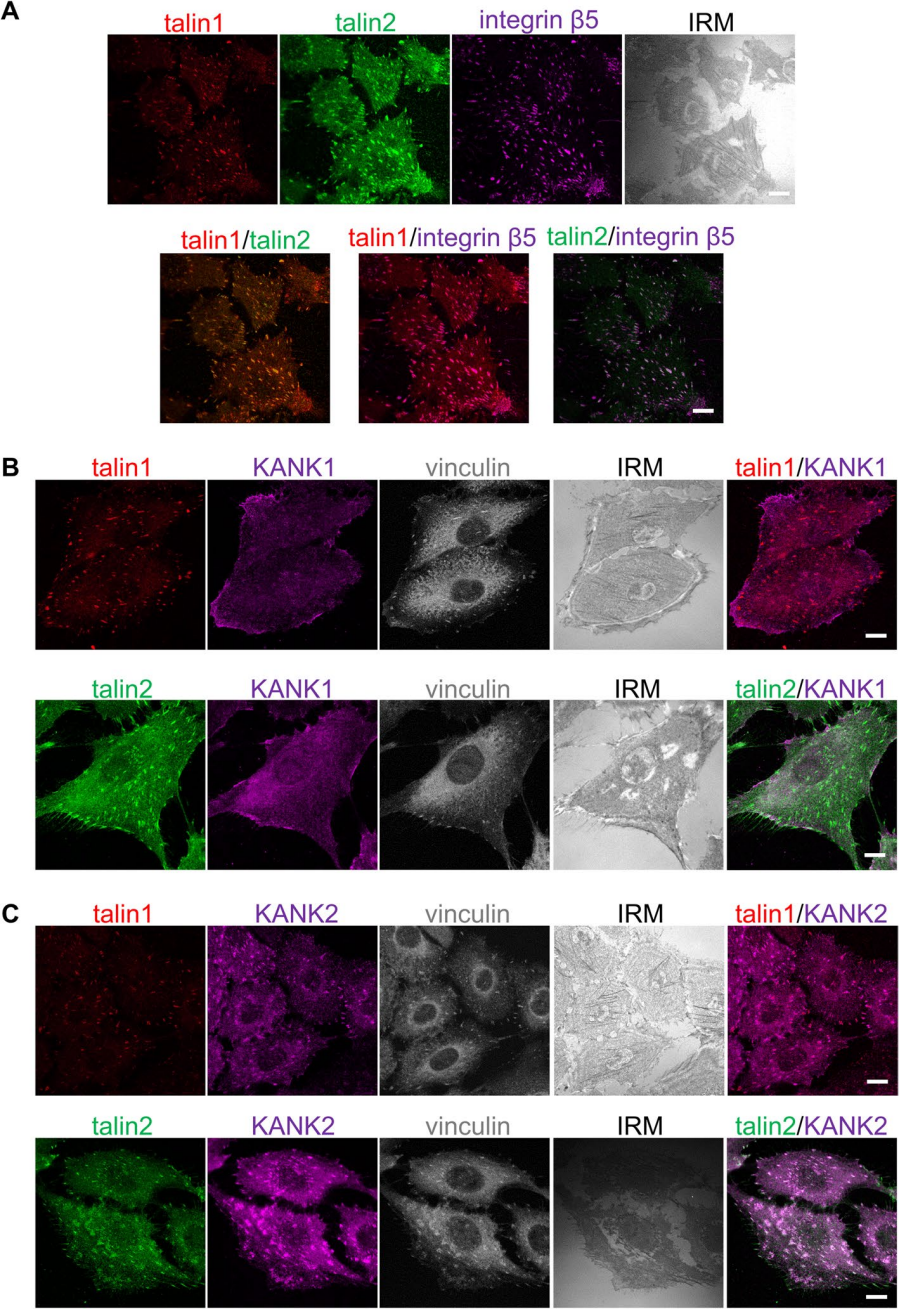
KANK2, unlike KANK1, is localized within αVβ5 FAs. **A** Talin1 and talin2 are localized within integrin αVβ5 FAs. Forty-eight hours after seeding MDA-MB-435S cells were methanol fixed, and stained with anti-talin1 antibody followed by Alexa-Fluor IgG1 555-conjugated antibody (red), anti-talin2 antibody followed by Alexa-Fluor IgG2b 488-conjugated antibody (green) and anti-integrin β5 antibody followed by Alexa-Fluor 647-conjugated antibody (magenta) and interference reflection microscopy (IRM) images were taken. **B**, **C** KANK2, unlike KANK1, localizes in talin1 and talin2-positive FAs. Forty-eight hours after seeding MDA-MB-435S cells were methanol fixed and stained with anti-talin1 or anti-talin2 antibody followed by Alexa-Fluor 546-conjugated antibody (red) or Alexa-Fluor 488-conjugated antibody (green) and anti-KANK1 or anti-KANK2 antibody followed by Alexa-Fluor 555-conjugated antibody or Alexa-Fluor 488-conjugated antibody (shown in magenta). Vinculin was visualized using conjugated anti-vinculin Alexa Fluor 647 antibody (shown in grey) and IRM images were taken. Analysis was performed using TCS SP8 Leica. Scale bar = 10 µm

To determine the location of talin isoforms within integrin αVβ5 FAs, we analyzed the distribution of talin1, talin2 and integrin β5 using IF. Figure 1A shows that both talin1 and talin2 colocalize with integrin β5, thus confirming MS data that both talins are constituents of integrin αVβ5 IACs [24]. Although KANK1 and KANK2 are similar in structure [22], they displayed different subcellular localizations in MDA-MB-435S cells. KANK1 was primarily restricted to the cell edge, in regions that were rich in talin1- and talin2-positive FAs (Fig. 1B). Conversely, KANK2 localized to talin1 and talin2-positive FAs both at the cell edge and in the center of the cell (Fig. 1C).

**Fig. 2:**
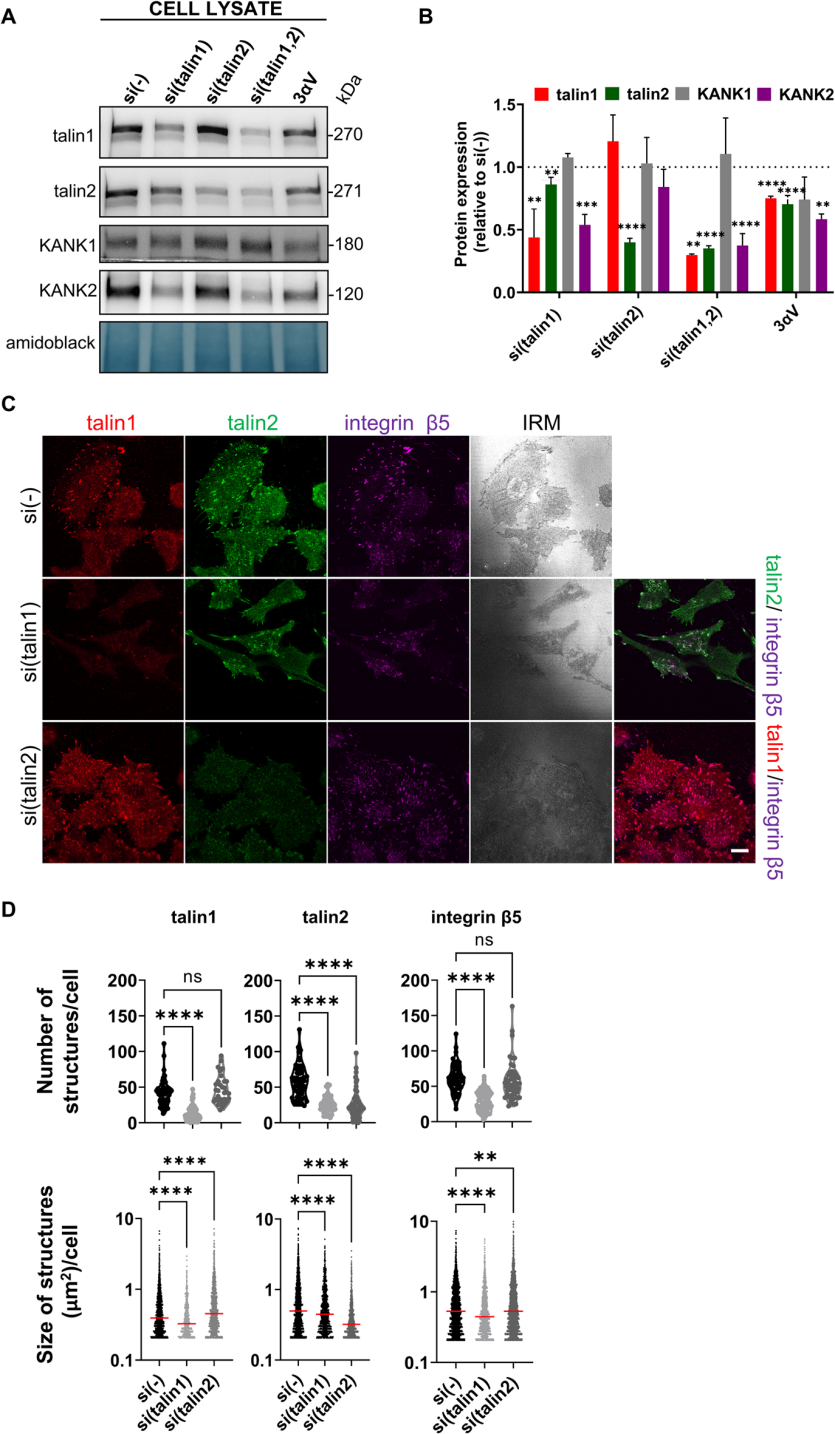
Talin1, unlike talin2, is necessary for formation of integrin αVβ5 FAs. **A** Talin1 knockdown, unlike talin2, reduces the level of KANK2 in whole cell lysates, while KANK1 level does not change upon either talin1 or talin2 knockdown. WB analysis of talin1, talin2, KANK1 or KANK2 in MDA-MB-435S cells transfected with either control, talin1, talin2 or a combination of talin1 and talin2-specific siRNAs and in the 3αV clone with decreased expression of integrin αV. Forty-eight hours after transfection total cell lysates were collected and WB analysis was performed. The results presented are representative of three independent experiments with similar results. **B** Quantification of data presented in (**A**). Histogram data are plotted as mean ± SD (*n* ≥ 3) relative to expression in MDA-MB-435S cells transfected with control siRNA that was set as 1 (indicated by a dotted line). Data were analyzed by one-way ANOVA with Dunnett’s multiple comparison. ns, not significant; * *P* < 0.05; ** *P* < 0.01; *** *P* < 0.001; **** *P* < 0.0001. **C** Knockdown of talin1 leads to disruption of αVβ5 FAs. Forty-eight hours after transfection with either control siRNA, talin1 or talin2-specific siRNA, MDA-MB-435S cells were methanol fixed, and stained with anti-talin1 antibody followed by Alexa-Fluor IgG1 555-conjugated antibody (red), anti-talin2 antibody followed by Alexa-Fluor IgG2b 488-conjugated antibody (green) and anti-β5 antibody followed by Alexa-Fluor 647-conjugated antibody (magenta) and IRM images were taken. Analysis was performed using TCS SP8 Leica. Scale bar = 10 µm. **D** Quantification of data presented in (**C**). Violin plots (number of structures/cell) and scatter plots with median marked in size (size of structures/cell) represents measurements of > 45 cells. Data were analyzed by one-way ANOVA with Dunnett’s multiple comparison. ns, not significant; * *P* < 0.05; ** *P* < 0.01; *** *P* < 0.001; **** *P* < 0.0001

We have previously shown that KANK2 is a key molecule linking integrin αVβ5 IACs to MTs, enabling the actin-MT crosstalk required for both sensitivity to MT poisons and cell migration. MDA-MB-435S cells also express KANK1, but it was not clear from our MS data whether there was a difference in KANK1 expression after integrin αV knockdown [24]. Therefore, we analyzed KANK1 expression in 3αV cells, obtained from MDA-MB-435S cells following stably decreased expression of integrin αV, by WB of total cell lysates, and found that its level was not significantly different from that in MDA-MB-435S cells (Fig. 2A, B; Additional file 1: Fig. S6). In addition, we performed knockdown of talin1 or talin2 and showed that neither KANK1 level (Fig. 2A,B; Additional file 1: Fig. S6) nor its localization changed (Additional file 1: Fig. S1A, B). Most significantly, knockdown of KANK1 did not alter the sensitivity of MDA-MB-435S cells to PTX (Additional file 1: Fig. S1C).

KANK2 expression was reduced in the 3αV clone compared to MDA-MB-435S cells [24] (Fig. 2A, B; Additional file 1: Fig. S6). To identify the isoform dependence of talin binding to KANK2, we analyzed the expression of KANK2 in MDA-MB-435S cells transiently transfected with talin1- or talin2-specific siRNA, using WB. Knockdown of talin 1 decreased the level of talin2 and KANK2, while knockdown of talin2 did not change the level of KANK2 (Fig. 2A, B; Additional file 1: Fig. S6). Simultaneous knockdown of both talins resulted in a comparable level of KANK2 as observed upon talin1 knockdown (Fig. 2A, B; Additional file 1: Fig. S6).

Next, the talin isoform dependency of MDA-MB-435S cell adhesion was analyzed by knockdown with control, talin1- or talin2-specific siRNA. Figure 2C shows that knockdown of talin1 (Fig. 2A, B; Additional file 1: Fig. S6), led to disruption of αVβ5 FAs and a simultaneous reduction of talin2-positive structures (Fig. 2C, D). This conclusion was supported by a reduction in the cell spreading area (Additional file 1: Fig. S2), with only a few remaining αVβ5 FAs containing talin1 (Fig. 2D first row), and shown by reduced expression of talin1 (Fig. 2A, B; Additional file 1: Fig. S6). It should be noted that after knockdown of talin1 there is some residual talin2 that colocalizes with integrin β5, which is very likely a part of RAs and whose presence in MDA-MB-435S cells was observed previously [5, 24]. Upon talin2 knockdown, colocalized integrin αVβ5 FAs and talin1 were still present (Fig. 2C) and cell spreading was not affected (Additional file 1: Fig. S2) despite the highly efficient knockdown of talin2 (Fig. 2A, B; Additional file 1: Fig. S6), demonstrated as disappearance of talin2-positive structures (Fig. 2C). This morphological analysis was supported by a quantitative assessment of the FA number, where talin1-, talin2- or β5-positive structures decreased upon talin1 knockdown, but the number of talin1- and β5-positive structures didn’t change upon talin2 knockdown (Fig. 2D first row). These data, together with decreased FA size upon talin1 knockdown, measured by analyzing talin1-, talin2- or β5-positive structures, and increased FA size upon talin2 knockdown, measured by analyzing talin1- and β5-positive structures but not by analyzing talin2-positive structures (Fig. 2D second row), indicates that talins are not functionally redundant.

**Fig. 3:**
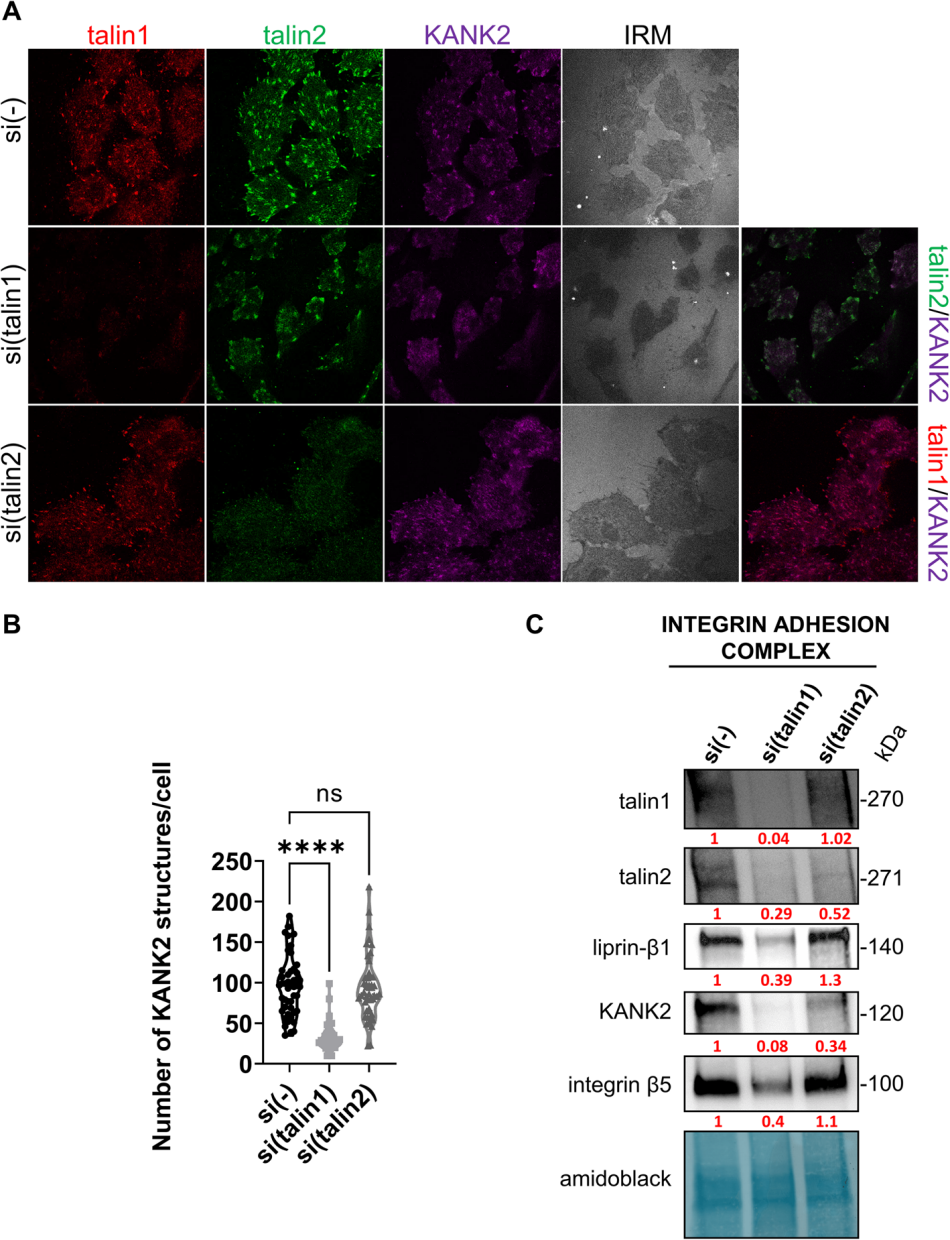
Talin2 functionally interacts with KANK2. **A** Knockdown of talin1, unlike talin2, leads to changes in KANK2 localization. Forty-eight hours after transfection with either control siRNA, talin1 or talin2-specific siRNA, MDA-MB-435S cells were methanol fixed and stained with anti-talin1 antibody followed by Alexa-Fluor IgG1 555-conjugated antibody (red), anti-talin2 antibody followed by Alexa-Fluor IgG2b 488-conjugated antibody (green), anti-KANK2 antibody followed by Alexa-Fluor 647-conjugated antibody (magenta) and IRM images were taken. Analysis was performed using TCS SP8 Leica. Scale bar = 10 µm. **B** Quantification of data presented in (**A**). Violin plot represents measurements of > 30 cells. Data were analyzed by one-way ANOVA with Dunnett’s multiple comparison. ns, not significant; * *P* < 0.05; ** *P* < 0.01; *** *P* < 0.001; **** *P* < 0.0001. **C** WB analysis of talin1, talin2, liprin-β1, KANK2 or integrin β5 in IACs isolated from MDA-MB-435S cells transfected with either control siRNA, talin1 or talin2-specific siRNA. Forty-eight hours after transfection, IACs were isolated and WB analysis was performed. The results presented are representative of two independent experiments yielding similar results. Numbers below WB scan represent expression of target proteins after talin1 or talin2 knockdown as relative to expression of target proteins in cell transfected with control siRNA set as 1

We next performed IF analysis of KANK2 following either talin1 or talin2 knockdown. Again, talin1 knockdown reduced cell spreading (Additional file 1: Fig. S2), with only a few remaining structures containing talin1, talin2 and KANK2 (Fig. 3A, B) consistent with their reduced level as measured by WB (Fig. 2A, B; Additional file 1: Fig. S6). Of note, we observed colocalization of talin2 and KANK2 remaining in structures lacking talin1 which is reminiscent of RAs [5, 24]. However, upon talin2 knockdown, there was no change in the expression (Fig. 2A, B; Additional file 1: Fig. S6) or the appearance of KANK2 which colocalizes with talin1 (Fig. 3A). A statistical analysis of the number of KANK2-positive structures per cell confirmed that only talin1 knockdown had a significant effect (Fig. 3B).

In conclusion, our data show that MDA-MB-435S cells in long term culture assemble FAs containing talin1, talin2 and KANK2 but not KANK1. In addition, these data indicate that talins are not functionally redundant. Namely, talin1 led to disruption of αVβ5 FAs and a simultaneous reduction of talin2- and KANK2 positive structures while reducing their total protein levels in the cell. In contrast, knockdown of talin2 did not affect either the protein levels or the localization of integrin αVβ5 FA, talin1, or KANK2.

### Talin isoform specific regulation of integrin αVβ5 and KANK in isolated IACs

Talins are located in FAs, while KANK2 bridges FAs to the CMSCs [14, 18, 20]. In our experimental system, the expression of CMSC components, including KANK2, depends on αVβ5 FAs, which is evident from MS analysis of isolated IACs upon knockdown of integrin αV [24], but also from WB and IF analysis upon talin1 knockdown, which leads to the reduction of KANK2 (Figs. 2A,B, 3A, B; Additional file 1: Fig. S6). Since talin2 knockdown results in an augmentation in FA size (Fig. 2D, second row), we hypothesized that KANK2 might bind talin2 and therefore, after talin2 knockdown, the connection between FAs and CMSC might be partially disrupted. Our attempts to demonstrate a direct link between talin1 or talin2 and KANK2 by coimmunoprecipitation failed. However, it was previously demonstrated, using in vitro fluorescence polarization assay, that both talins (1 and 2) can bind both KANKs (1 and 2) through interaction of KN domain with talin R7 domain [14]. Therefore, we sought to demonstrate a functional interaction between talins and KANK2 within αVβ5 FAs in MDA-MB-435 s cells. To test this, we performed knockdown of either talin1 or talin2, crosslinked and isolated IACs and analyzed the levels of talin1, talin2, integrin β5, KANK2 and one of the main components of CMSC, liprin-β1. As expected, knockdown of talin1 reduced the levels of integrin β5, talin2, KANK2 and liprin-β1 while knockdown of talin2 did not affect levels of β5 or talin1 from FAs (Fig. 3C, Additional file 1: Fig. S7), which is in line with our previous data (Fig. 2D). However, despite the fact that upon talin2 knockdown, KANK2 did not change its localization (Fig. 3A) or expression level in total cell lysate (Fig. 2A ,B; Additional file 1: Fig. S6), talin2 knockdown reduced the level of crosslinked KANK2 in samples of isolated IACs (Fig. 3C, Additional file 1: Fig. S7), suggesting that talin2 and KANK2 might be interaction partners. In other words, talin2 knockdown disrupted the link between FA and CMSC. The level of liprin-β1 did not change upon talin2 knockdown (Fig. 3C, Additional file 1: Fig. S7) indicating that the formation of CMSCs does not depend on talin2. This conclusion is further supported by monitoring the localization of liprin-β1, upon talin2 or KANK2 knockdown. Knockdown of talin2 did not change the localization of liprin-β1 (Additional file 1: Fig. S3A). However, knockdown of KANK2 reduced the amount of liprin-β1 (Additional file 1: Fig. S3B). Therefore, we conclude that the binding of KANK2 to the CMSC is essential for its stabilization and specific localization near FAs.

### Talin isoform specific regulation of the actin and MT cytoskeleton

**Fig. 4:**
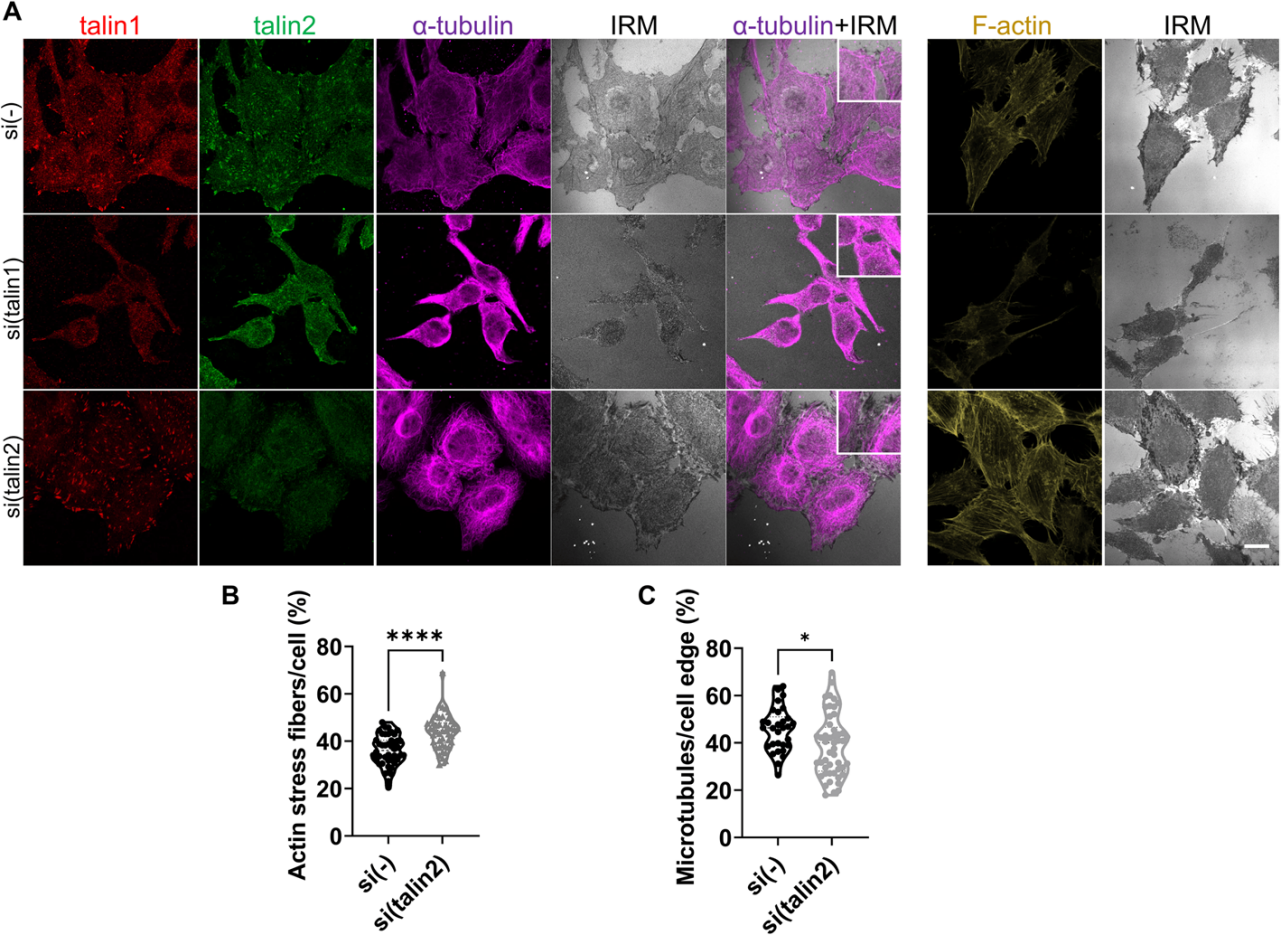
Talin1 or talin2 knockdown affects actin and MT cytoskeleton*.* **A** Visualization of α-tubulin and F-actin upon knockdown of talin1 or talin2. Forty-eight hours after transfection with either control, talin1 or talin2-specific siRNA, MDA-MB-435S cells were fixed with methanol (for α-tubulin visualization) or PFA followed by permeabilization with Triton X-100 (for F-actin visualization), and stained with anti-talin1 antibody followed by Alexa-Fluor IgG1 555-conjugated antibody (red), anti-talin2 antibody followed by Alexa-Fluor IgG2b 488-conjugated antibody (green), anti-α-tubulin antibody followed by Alexa-Flour 647-conjugated antibody (magenta) and IRM images were taken. For F-actin visualization cells were incubated with Alexa-Flour 488 conjugated phalloidin (shown in gold). Analysis was performed using TCS SP8 Leica. Scale bar = 10 µm. **B**, **C** Quantification of data presented in (**A**). Violin plots represents measurements of > 30 cells. Data were analyzed by unpaired Student’s *t*-test. ns, not significant; *P < 0.05; **P < 0.01; ***P < 0.001; ****P < 0.0001

MDA-MB-435S cells contain a high level of stress fibers [37]. KANK2 knockdown in MDA-MB-435S cells does not alter cell size or stress fiber level, but alters MT appearance [24]. We therefore analyzed changes in the actin and MT cytoskeleton upon talin1 or talin2 knockdown using IF. Knockdown of talin1 led to reduced cell spreading area (Additional file 1: Fig. S2) and a significant loss of stress fibers (Fig. 4A). Knockdown of talin2 did not alter cell area (Additional file 1: Fig. S2), but increased the level of stress fibers (Fig. 4A) confirmed by the quantification of confocal images (Fig. 4B). We also observed a complete breakdown of the MT network upon talin1 knockdown (Fig. 4A) which is in line with the disruption of integrin αVβ5 FAs (Fig. 2C). Upon talin2 knockdown, the MT network was still present, but was denser than the control (Fig. 4A) and resembled the MT network upon KANK2 knockdown [24]. We also performed quantitative MT image analysis within 5 µm from the cell edge and showed a slightly decreased MT content (Fig. 4C). These data provide further evidence that talin2 and KANK2 are potential interaction partners.

### Breaking the link between CMSCs and FAs leads to increased velocity of microtubule growth

As the results above suggest that KANK2 might be an interaction partner of talin2, thus linking the integrin αVβ5 FAs to the CMSCs, we hypothesized that the mechanism of PTX sensitization might be changes in MT dynamics. In order to test this hypothesis, we measured the velocity of MT growth upon knockdown of talins and KANKs. To visualize changes in the velocity of MT growth, we isolated a population of MDA-MB-435S cells and 3αV cell clone transfected with fluorescent EB3 (435S-EB3 and 3αV-EB3). Since fluorescent EB3 (EB3-Dendra2) binds to the growing tip of MTs, growing MTs could be followed during time-lapse live cell microscopy [39]. The 435S-EB3 and 3αV-EB3 cell populations retained their initial characteristics after isolation (Additional file 1: Fig. S4). The 3αV-EB3 cells expressed lower amounts of talin1, integrin β5 and KANK2 compared to 435S-EB3 (Additional file 1: Fig. S4A, B) and were more sensitive to PTX compared to 435S-EB3 cells (Additional file 1: Fig. S4C).

**Fig. 5:**
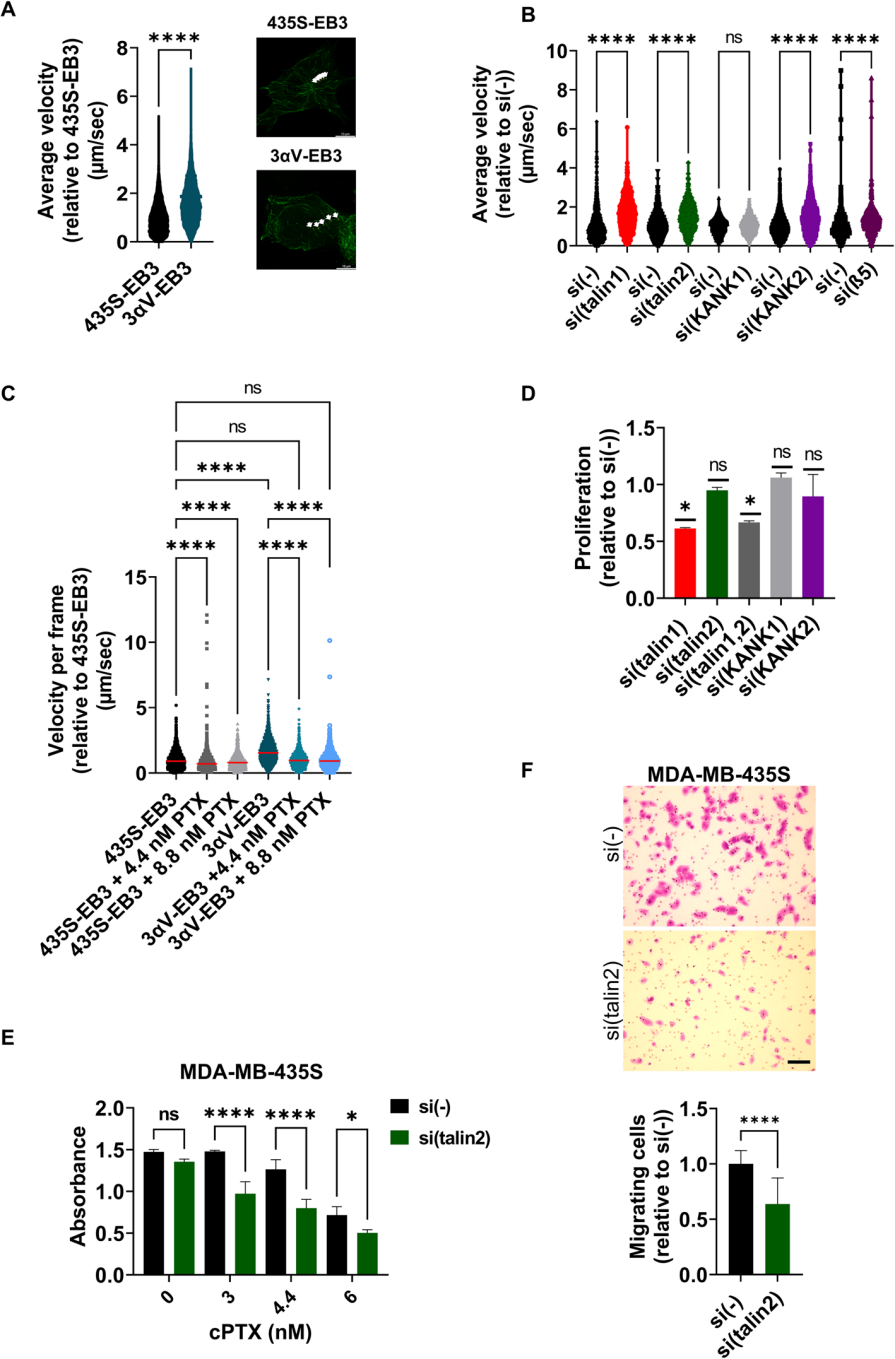
Disruption of crosstalk between CMSCs and FAs leads to increased velocity of microtubule growth and increased sensitivity to PTX. **A** Knockdown of αV integrin subunit increases velocity of microtubule growth. Quantification of time-lapse live cell microscopy data of 435S-EB3 cells and 3αV-EB3 cell clone with decreased expression of integrin αV. Violin plot represents measurements of > 450 analyzed microtubules, (*n* = 3) relative to velocity of MT growth in MDA-MB-435S cells that was set as 1. Data were analyzed by unpaired Student’s *t*-test. ns, not significant; *P < 0.05; **P < 0.01; ***P < 0.001; ****P < 0.0001. Still images of Additional file 2 : Movie S1 and Additional file 3: S2 represent tracking of one microtubule tip in 104 s through five frames. (B) 435S-EB3 cells transfected with either talin1, talin2, KANK2 or β5-specific siRNA, but not KANK1 siRNA, showed a significant increase in velocity of MT growth compared to cells transfected with control siRNA. Quantification of time-lapse live cell microscopy data. Violin plot represents measurement of > 250 analyzed microtubules, (*n* ≥ 2) relative to velocity of MT growth in MDA-MB-435S cells transfected with control siRNA that was set as 1. Data were analyzed by one-way ANOVA with Šídák’s multiple comparisons test. ns, not significant; *P < 0.05; **P < 0.01; ***P < 0.001; ****P < 0.0001. **C** PTX treatment decreases MT growth velocity much stronger in 3αV-EB3 cells compared to 435S-EB3 cells. Quantification of time-lapse live cell microscopy data of 435S-EB3 cells and 3αV-EB3 clone upon treatment with equitoxic and equimolar concentrations of PTX. Scatter plot with median marked in red represents measurements of > 200 analyzed microtubules, (*n* = 3) relative to velocity of MT growth in MDA-MB-435S cells that was set as 1. Data were analyzed by one-way ANOVA with Šídák’s multiple comparisons test. ns, not significant; *P < 0.05; **P < 0.01; ***P < 0.001; ****P < 0.0001. **D** Cell proliferation decreases upon knockdown of talin1, but not talin2, KANK1 or KANK2. Cell proliferation was measured using ClickIT EdU assay upon transfection with either control, talin1, talin2, combination of talin1 and talin2, KANK1 or KANK2-specific siRNA. Histogram represents measurements of > 30 cells, plotted as mean ± SD (*n* = 2). Data were analyzed by one-way ANOVA with Dunnett’s multiple comparison. ns, not significant; *P < 0.05; **P < 0.01; ***P < 0.001; ****P < 0.0001. **E** Talin2 knockdown increases sensitivity to PTX in MDA-MB-435S cells. Twenty-four hours upon transfection, cells were seeded in 96-well plates and 24 h later treated with different concentrations of PTX. Cytotoxicity was measured by MTT assay. Data were analyzed by two-way analysis of variance (ANOVA) with Šídák’s multiple comparisons test, with a single pooled variance. ns, not significant; *P < 0.05; **P < 0.01; ***P < 0.001; ****P < 0.0001. (*n* = 3). **F** Talin2 knockdown decreases migration in MDA-MB-435S cells. Serum starved (24 h) cells, transfected previously with either control or talin2-specific siRNA were seeded in Transwell cell culture inserts and left to migrate for 22 h toward serum. Cells on the underside of the inserts were stained with crystal violet, photographed, and counted. Scale bar = 100 µm. **F** Histogram data represents averages of five microscope fields of three independently performed experiments, plotted as mean ± SD. Data were analyzed by unpaired Student’s *t*-test. ns, not significant; *P < 0.05; **P < 0.01; ***P < 0.001; ****P < 0.0001

Using live cell imaging of fluorescent EB3, we showed that the velocity of MT plus end growth in 3αV-EB3 was approximately 1.6 times faster than in 435S-EB3 cells (Fig. 5A; Additional file 8: Fig. S8; Additional file 2: Movie S1, Additional file 3: Movie S2). Knockdown of talin1, talin2, KANK2 or integrin β5 had the same effect, while knockdown of KANK1 had no effect (Fig. 5B; Additional file 8: Fig. S8; Additional file 2: Movie S1; Additional file 3: Movie S2; Additional file 4: Movie S3, Additional file 5: Movie S4, Additional file 6: Movie S5, Additional file 7: Movie S6). We conclude that knockdown of integrin αV, integrin β5, talin1, talin2 or KANK2 interferes with either integrin αVβ5 FA formation (integrin αV, integrin β5 or talin1) or breaks the link between the MT cytoskeleton and integrin αVβ5 FAs (talin2 or KANK2). These results therefore further support the conclusion that talin2-KANK2 interaction is functional in connecting FAs and CMSCs and that its loss leads to changes in MT dynamics.

### The increased sensitivity to paclitaxel correlates with increased velocity of microtubule growth

PTX is among the most widely used chemotherapeutic drugs which binds to and stabilizes MTs. PTX causes concentration-dependent inhibition of MT dynamics and perturbation of mitosis [40]. Considering that 435S-EB3 and 3αV-EB3 cells show different sensitivity to PTX and different velocities of MT growth, we exposed both cells to an equitoxic or equimolar dose of PTX and measured the velocity of MT growth using time-lapse imaging. A decrease in the velocity of MT growth was observed in both 435S-EB3 and 3αV-EB3 cells with both concentrations of PTX with respect to the nontreated control (Fig. 5C). However, in 3αV-EB3 cells, which demonstrated a 1.6-fold increased velocity compared to 435S-EB3 cells (Fig. 5A), the reduction was much more pronounced (Fig. 5C).

Since we have previously shown that KANK2 knockdown sensitizes cells to PTX [24], and have demonstrated here a functional link between talin2 and KANK2 and their effect on MT dynamics, we tested the effects of talin2 knockdown on sensitivity of MDA-MB-435S cells to PTX by measurement of cell survival following the treatment with PTX. It has to be emphasized that neither talin2 nor KANK2 knockdown altered cell proliferation, thus indicating that the observed sensitization is the result of altered MT dynamics. Cell proliferation was affected only by talin1 knockdown (Fig. 5D), consistent with the fact that it leads to the destruction of FAs. Talin2 knockdown increased sensitivity to PTX compared to cells transfected with control siRNA (Fig. 5E) and reduced cell migration (Fig. 5F). Since cell migration is a process driven by the cytoskeleton [41] it was not surprising that talin2 knockdown also reduced MDA-MB-435S cell migration as was observed for KANK2 knockdown [24].

In conclusion, the data presented here support a functional interaction between KANK2 and talin2 that maintains the link between FAs and CMSCs, affecting actin-MT crosstalk and regulating sensitivity to MT poison PTX and cell migration.

## Discussion

MTs can affect FA dynamics and functions through trafficking and signaling functions, but it is also clear that there is a physical and functional link between MTs and FAs. The importance of this actin-MT crosstalk has been recognized in several physiological processes, survival and migration being some of them [41, 42]. In this paper, we have shown that the loss of interaction between αVβ5 FAs and MTs leads to increased sensitivity to PTX and that the mechanism by which this occurs is a change in the velocity of MT growth. Our results identify, in melanoma cell line MDA-MB-435S, talin2 from integrin αVβ5 FAs and KANK2 from CMSCs, as functional partners regulating MT dynamics and sensitivity to PTX. Specifically, stable integrin αV knockdown in 3αV-EB3 cells, which reduces talin1, talin2 and KANK2 levels, increased the velocity of MT growth and sensitivity to PTX as compared to 435S-EB3 cells. Importantly, upon PTX treatment, a reduction of the velocity of MT growth is much more pronounced in 3αV-EB3 than in 435S-EB3 cells thus correlating to the sensitivity to PTX. In MDA-MB-435S cells, knockdown of talin2 or KANK2 mimics the effect of integrin αV knockdown, i.e. increased velocity of MT growth and increased sensitivity to PTX, thus providing evidence that KANK2 functionally interacts with talin2.

Because knockdown of talin1 disrupts and effectively removes all links between the proteins within and associated to αVβ5 FAs, while knockdown of talin2 does not affect the formation or number of αVβ5 FA, and because, in our hands, co-immunoprecipitation experiments between talins and KANK2 failed, we couldn’t conclude which talin physically binds KANK2. However, we show that talin2 and KANK2 functionally interact by analyzing isolated IACs after talin2 knockdown in which the level of cross-linked KANK2 is significantly reduced. It has been shown in vitro that talin1 and talin2 can bind KANK2 through interaction with talin R7 domain [14]. However, to our knowledge, direct link between endogenous talins and KANK2 was not shown using co-immunoprecipitation. The localization of KANK1 [14] or KANK2 [24] within CMSC has been demonstrated but the cause of these differences is not known. The functional interaction of talin2 and KANK2 is very likely cell type specific affected by unknown factor/s that should be investigated in the future.

The knockdown of integrin αV [37], KANK2 [24] and talin2 (this work) not only increased sensitivity to PTX but also decreased cell migration. Similarly, in HeLa cells, the loss of KANK1 reduced cell motility [14] while in cultured mouse podocytes depletion of both, KANK1 or KANK2, resulted in reduced migration [43]. Conversely, in mouse fibroblasts KANK2 does not localize within CMSC but to the lateral border of FAs (FA belt) where it activates talin, reduces force transduction across integrins and induces central adhesion formation. This adhesion sliding correlates with reduced cell migration speed i.e. KANK2 depleted mouse fibroblast cells showed higher migration velocities [20]. Therefore, KANK proteins play a dual role in the cell: they can play a role in MT targeting [14] or in force transmission, but these KANK2 complexes function separately [20]. We can assume that KANK localization and interaction partners potentially determine the way it will affect cell migration.

We showed in our MDA-MB-435S cells that KANK1 is not a part of the CMSC associated with integrin αVβ5 FAs because KANK1 level does not change after knockdown of either talin1, talin2 or integrin αV, nor does KANK1 knockdown affect cell sensitivity to PTX or change the velocity of MT growth. KANK1 function and potential interaction partners should be further investigated.

Currently little is known about the isoform dependence of talin-KANK binding. In HeLa cells KANK1 binds to talin1 and controls cortical organization of CMSC components [14] while in the fibrosarcoma cell line HT1080 KANK1 or KANK2 knockdown mimicked the effects of nocodazole-induced MT depolymerization i.e. uncoupling MTs from adhesion complexes [44]. KANK2 in mouse fibroblasts is present in mature integrin α5β1 FAs where it regulates force transmission in a CMSC-independent manner [20].

Talin1 and talin2 mRNAs display different tissue distributions and a reciprocal relationship between their expression [45]. Even though the isoforms share 76% protein sequence their role in cells is not fully redundant [46–48] and the mechanism of interplay of the isoform expression is still not known [26]. Our results support cell-specific and αVβ5 integrin-specific ratio of talin1 and talin2 in FAs. We have previously shown that integrin αVβ5 FAs, used preferentially for adhesion in MDA-MB-435S cells, contain both talins [24]. Interestingly, using the same cell line, Qi and colleagues [46] showed that talin1 is required for small FA formation, whereas talin2 is responsible for larger FA assembly. However, they plated cells on fibronectin and therefore αVβ5 may not have been the primary receptor used by cells. In several cell types talin1 was concentrated in peripheral FAs while talin2 was observed in both FAs and fibrillar adhesions [49]. In our case both talin isoforms, 1 and 2, were present in αVβ5 FAs. We showed that talin1 has a role in formation of αVβ5 FAs since knockdown of talin1, unlike talin2, led to the disruption of αVβ5 FAs. It would follow that this disruption would result in a decrease of other proteins within the FAs. Since we have previously shown that talin2 and KANK2 are also a part of the integrin αVβ5 FAs [24], it was to be expected that knockdown of talin1 would lead to a decrease in the level of talin2 and KANK2. Besides, that means that talin2 alone is not sufficient for successful integrin αVβ5 FA formation, which is consistent with the fact that knockout of talin1, but not talin2, is lethal in mice [27, 28]. This conclusion is supported by the fact that in our MDA-MB-435S cells the proliferation was strongly affected by talin1 knockdown (down to 60%) but not upon talin2 knockdown (down to 95%). It should be noted that talin1 cannot replace the role of talin2 in maintaining actin-MT crosstalk, because after talin2 knockdown in IAC isolates KANK2 levels are reduced and the velocity of MT growth is increased, thus indicating uncoupling of MTs from FAs. Together, these results confirm that talins are not functionally redundant.

In our MDA-MB-435S cells the size of talin1 and β5-positive structures upon talin2 knockdown increased, without affecting the number of αVβ5 FAs, indicating the change in FA dynamics. The knockdown of the other functional partner involved in actin MT-crosstalk, KANK2, also slightly increased talin2-positive structures (Additional file 1: Fig. S5) thus supporting a talin2-KANK2 functional linkage. In addition, we observed an increased amount of stress fibers upon talin2 knockdown which is not the consequence of talin1 replacing talin2, since WB analysis of talin1 in either total cell lysates or IACs showed that its level did not change upon talin2 knockdown. Conversely, KANK2 knockdown did not alter the amount of stress fibers [24]. The absence of changes in amount of stress fibers upon KANK2 knockdown still does not exclude functional involvement of KANK2 in actin cytoskeleton dynamics. Indeed, it has been shown in the human fibrosarcoma cell line HT1080 that uncoupling of FAs and CMSCs, by depleting KANKs or disrupting MT stability, leads to dispersion of CMSCs and augmentation of FAs. The release of the RhoA GEF ARHGEF2/GEF-H1 from MTs resulted in increased RhoA-mediated actomyosin contractility, reinforcement of the stress fiber-associated FAs and decreased FA turnover [44].

KANK proteins are generally localized to the periphery of FAs [14, 20] and complex stability measurements combined with cell biological experiments suggested that shear-force stretching promotes KANK1 localization to the periphery of FAs [21]. Since talin2 knockdown did not affect the formation and number of αVβ5 FAs we assume that in our cell model FAs are formed preferentially through involvement of talin1 while talin2 is recruited to the outer rim of FAs later, enabling the link to KANK2 protein from CMSC and establishing actin-MT crosstalk. The talins are mehanotransducers due to their ability to undergo force dependent structural rearrangements [26]. It has been shown in vitro that cyclin-dependent kinase-1 (CDK1) regulates IACs through binding within the R8 domain of talin1 and phosphorylation at the end of R7 rod domain leading to significant reduction in binding of KANK to R7 [50]. We hypothesize that in our cell model, talin1, shown to be engaged in IAC formation, is likely phosphorylated by CDK1 and therefore has a low KANK binding affinity. Instead, talin2, which we have shown is not essential for FA formation, is the one sterically accessible and maintains an affinity to KANK2.

We have shown that talin2 knockdown, which disconnects FAs from CMSCs, does not affect either KANK2 or liprin-β1 localization. Nevertheless, upon KANK2 knockdown, the liprin-β1 signal was dispersed and almost lost. Indeed, the clustering of CMSC components depends on interactions of its components, primarily KANK and liprins α1 and β1 and possibly other interactions [15, 18, 19, 51]. Our results are in line with results obtained by Bouchet and colleagues [14] in HeLa cell model in which the localization of CMSC clusters depends on the KANK1-liprin-β1 connection.

Integrin expression is frequently disturbed in tumor cells and therefore integrins represent attractive therapeutic targets for arresting tumor growth, reducing resistance to chemo-or radiotherapy, or attenuating invasion and metastasis [52]. We have previously identified, in melanoma MDA-MB-435S cells, integrin αV as a potential therapeutic target whose knockdown increased sensitivity to PTX and reduced metastasis [24, 37]. However, targeted integrin therapy has proven to be ineffective in the clinic [52, 53]. We therefore suggest here, that besides KANK2, which we already previously identified as a protein linked to integrin αVβ5-mediated sensitivity to PTX [24], talin2 might also be a potential therapeutic target because its knockdown improves PTX therapy and reduces metastasis, and may have a more uniform response than targeting integrins. Disruption of a functional link between talin2 and KANK2 isoforms, either by KANK2 or talin2 knockdown, alters MT dynamics, i.e., increases the velocity of MT growth, thereby increasing sensitivity to PTX and decreasing migration. Indeed, few data from the literature confirm that talin2 is a potential target for tumor therapy because of its role in cell migration, invasion and metastasis. It has previously been shown that talin2 binds much more strongly to the β1-integrin tail than talin1 and that interaction of talin2 with integrins is required to generate traction force, which in turn drives invadopodium-mediated matrix degradation [46, 54]. In the breast cancer cell line MDA-MB-231, the loss-of-function of talin1 has been shown to increase chemosensitivity to docetaxel [55] while talin2 knockdown inhibited growth, migratory capacity and invasiveness and promoted apoptosis [54, 56]. Both talin1 and talin2 correlate with the malignancy potential of the human hepatocellular carcinoma MHCC-97 L cells [57]. Conversely, talin2 is downregulated in clear cell renal cell carcinoma (ccRCC) tissues and cells, and its overexpression in ccRCC cell lines inhibits cell growth and metastasis [58]. It remains to be discovered which factors influence the role of talin2 in tumors.

**Fig. 6:**
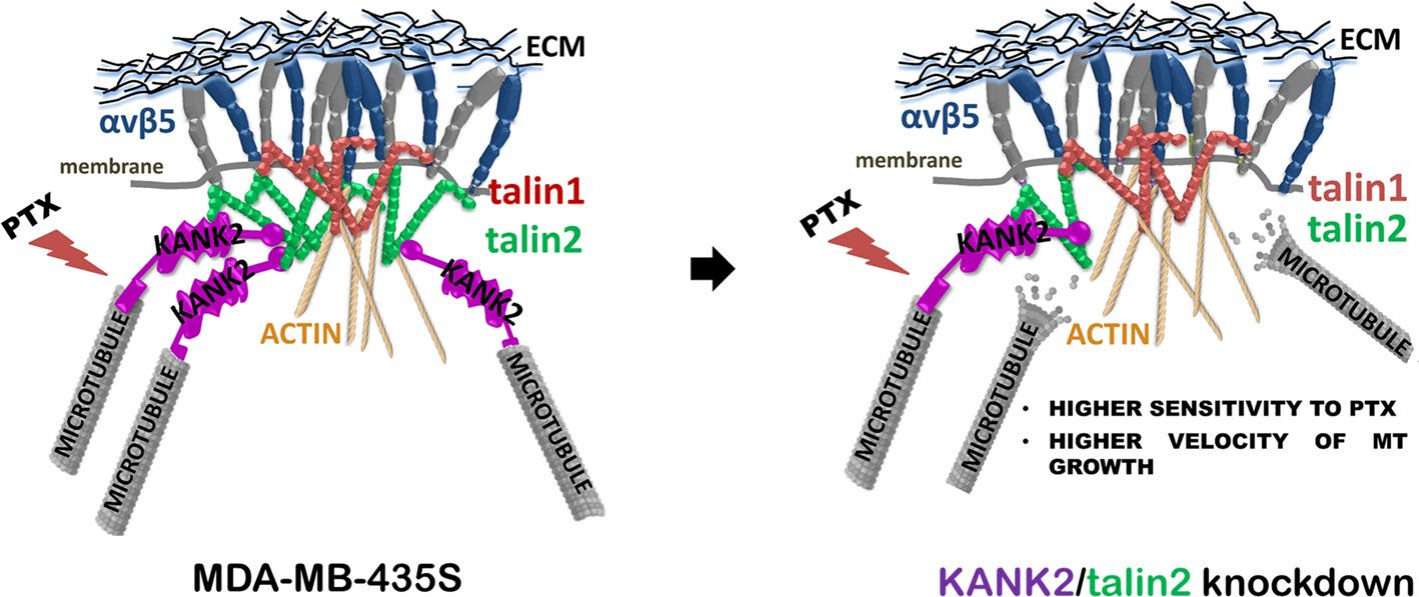
Schematic representation of the functional link between talin2 from αVβ5 FAs and KANK2 from CMSC. Disruption of the link, either by KANK2 or talin2 knockdown, results in increased velocity of MT growth, increased sensitivity to PTX and decreased migration of MDA-MB-435S cells

In conclusion, we show in melanoma cell line MDA-MB-435S the different subcellular localizations of KANK1 and KANK2, different roles of talin1 and talin2, and functional link between talin2 and KANK2 affecting MT dynamics, sensitivity to PTX and cell migration (Fig. 6). The different subcellular localizations of KANK isoforms 1 and 2 likely are caused by yet unknown interaction partners which should be further investigated.

## Supplementary Information

**Additional file 1: Fig. S1.** KANK1 localization is not affected by talin1 or talin2 knockdown. (A, B) Talin2, but not talin1, knockdown slightly affects KANK1 appearance. Forty-eight hours after transfection with either control, talin1 or talin2-specific siRNA, MDA-MB-435S cells were methanol fixed and stained with anti-talin1 or anti-talin2 antibody followed by Alexa-Fluor 546-conjugated antibody (red) or Alexa-Fluor 488-conjugated antibody (green), respectively. KANK1 was further visualized by anti-KANK1 antibody followed by Alexa-Fluor 555-conjugated antibody or Alexa-Fluor 488-conjugated antibody (shown in magenta). Finally, vinculin was visualized using conjugated anti-vinculin Alexa Fluor 647 antibody (shown in grey) and IRM images were taken. Analysis was performed using TCS SP8 Leica. Scale bar = 10 µm. (C) KANK1 knockdown does not affect sensitivity to PTX. Sensitivity of cells transfected with either control or KANK1-specific siRNA to PTX was measured by MTT assay. Twenty-four hours upon transfection, cells were seeded in 96-well plates and 24 h later treated with different concentrations of PTX. Data were analyzed by two-way analysis of variance (ANOVA) with Šídák’s multiple comparisons test, with a single pooled variance; ns, not significant; *P < 0.05; **P < 0.01; ***P < 0.001; ****P < 0.0001. **Fig. S2.** Cell area decreases upon knockdown of talin1, but not talin2. Quantification of data presented in Fig. 2. Violin plot represents measurements of > 30 cells (*n* = 2). Data were analyzed by one-way ANOVA with Dunnett’s multiple comparison. ns, not significant; *P < 0.05; **P < 0.01; ***P < 0.001; ****P < 0.0001. **Fig. S3.** The CMSC protein liprin-β1 loses its organization upon KANK2, but not upon talin2 knockdown. (A, B) Forty-eight hours after transfection with talin2 or KANK2-specific siRNA, MDA-MB-435S cells were fixed with methanol and stained with anti-liprin-β1 antibody followed by Alexa-Flour 546-conjugated antibody, anti-talin2 antibody followed by Alexa-Fluor IgG2b 488-conjugated antibody (green) and anti-KANK2 antibody followed by Alexa-Flour 647-conjugated antibody and IRM images were taken. Analysis was performed using TCS SP8 Leica. Scale bar = 10 µm. **Fig. S4.** Verification of the MDA-MB-435S and 3αV cell model expressing fluorescently labelled EB3. (A, B) Clone 3αV-EB3 shows decreased expression of talin1, integrin β5 and KANK2 as compared to MDA-MB-435S-EB3 cells. Forty-eight hours after seeding cells were methanol fixed and stained with anti-talin1 followed by Alexa-Fluor 546-conjugated antibody (red), anti-KANK2 antibody or anti-β5 antibody followed by Alexa-Fluor 647-conjugated antibody (magenta) and IRM images were taken. Analysis was performed using TCS SP8 Leica. Scale bar = 10 µm. (C) Clone 3αV-EB3 demonstrates increased sensitivity to PTX as compared to parental MDA-MB-435S-EB3 cells. Twenty-four hours upon seeding in 96-well plates cells were treated with different concentrations of PTX. Cytotoxicity was measured by MTT assay. Data were analyzed by two-way analysis of variance (ANOVA) with Šídák’s multiple comparisons test, with a single pooled variance; ns denotes not significant; * *P* < 0.05; ** *P* < 0.01; *** *P* < 0.001; **** *P* < 0.0001 (*n* = 3). **Fig. S5.** Knockdown of KANK2 increases talin2-positive FAs size. (A) Forty-eight hours after transfection with either control or KANK2-specific siRNA, MDA-MB-435S cells were methanol fixed and stained with anti-talin2 antibody followed by Alexa-Fluor IgG2b 488-conjugated antibody (green) and anti-KANK2 antibody followed by Alexa-Fluor 647-conjugated antibody (magenta) IRM images were taken. (B) Quantification of data presented in (a). Scatter plot with median marked in red represents measurements of ≥ 30 cells, (*n* = 2). Data were analyzed by unpaired Student’s *t*-test. ns, not significant; *P < 0.05; **P < 0.01; ***P < 0.001; ****P < 0.0001. **Fig. S6.** Full images of the blots in Fig. 2A. Images were obtained using Uvitec Alliance Q9 mini, which directly scanned membranes developed with ECL reagents. **Fig. S7.** Full images of the blots in Fig. 3C. Images were obtained using Uvitec Alliance Q9 mini, which directly scanned membranes developed with ECL reagents.

**Additional file 2: Movie S1.** Time-lapse live cell microscopy of MDA-MB-435S cells with fluorescent EB3 (435S-EB3 cells) used for measurement of velocity of MT growth.

**Additional file 3: Movie S2.** Time-lapse live cell microscopy of clone 3αV cells with fluorescent EB3 (3αV-EB3 cells) used for measurement of velocity of MT growth.

**Additional file 4: Movie S3.** Time-lapse live cell microscopy of MDA-MB-435S cells with fluorescent EB3 (435S-EB3 cells) transfected with control siRNA used for measurement of velocity of MT growth.

**Additional file 5: Movie S4.** Time-lapse live cell microscopy of MDA-MB-435S cells with fluorescent EB3 (435S-EB3 cells) transfected with talin1-specific siRNA used for measurement of velocity of MT growth.

**Additional file 6: Movie S5.** Time-lapse live cell microscopy of MDA-MB-435S cells with fluorescent EB3 (435S-EB3 cells) transfected with talin2-specific siRNA used for measurement of velocity of MT growth.

**Additional file 7: Movie S6.** Time-lapse live cell microscopy of MDA-MB-435S cells with fluorescent EB3 (435S-EB3 cells) transfected with KANK2-specific siRNA used for measurement of velocity of MT growth.

**Additional file 8: Fig. S8.** Still images of Additional file 2–Additional file 8: Movie S1–S6. Images were obtained using Image J manual tracking tool. Each arrow represent position of one microtubule tip through 104 s. Images were captured every 26 s.

**Additional file 9: Table S1.** List of used antibodies and dyes.

## Data Availability

All relevant data could be obtained from the corresponding author.

## Abbreviations

ECM: Extracellular matrix
IAC: Integrin adhesion complex
FA: Focal adhesion
RA: Reticular adhesion
MT: Microtubule
CMSC: Cortical microtubule stabilizing complex
PMAP: Plasma membrane-associated platform
ELKS: Proteins rich in amino acids E, L, K and S
KIF21A: Kinesin family member KIF21A
CLASP: CLIP-associating protein
KANK: KN motif and ankyrin repeat domain
MS: Mass spectrometry
KN: KANK N-terminal
CC: Coiled-coil
ANKRD: Ankyrin repeat domain
PTX: Paclitaxel
EB3: End-binding protein 3
SDS-PAGE: Sodium dodecyl sulphate–polyacrylamide-gel electrophoresis
WB: Western blotting
HEPES: Hydroxyethylpiperazine ethanesulfonic acid
DTBP: Dimethyl 3,3’-dithiobispropionimidate, Wang and Richard’s reagent
MTT: 3-((4,5-Dimethylthiazol-2-yl)-2,5- diphenyltetrazolium bromide
IRM: Interference reflection microscopy
IF: Immunofluorescence analysis
CDK1: Cyclin-dependent kinase-1
ANOVA: One-way analysis of variance
ccRCC: Clear cell renal cell carcinoma
