# Supplementary material for: Talin2 and KANK2 functionally interact to regulate microtubule dynamics, paclitaxel sensitivity and cell migration in the MDA-MB-435S melanoma cell line
Source: Cell Mol Biol Lett. 2023 Jul 17;28:56. doi: 10.1186/s11658-023-00473-6 (PMC10353188; doi:10.1186/s11658-023-00473-6)
Supplement: Supplementary file 1 — Additional file 1: Fig. S1. KANK1 localization is not affected by talin1 or talin2 knockdown. (A, B) Talin2, but not talin1, knockdown slightly affects KANK1 appearance. Forty-eight hours after transfection with either control, talin1 or talin2-specific siRNA, MDA-MB-435S cells were methanol fixed and stained with anti-talin1 or anti-talin2 antibody followed by Alexa-Fluor 546-conjugated antibody (red) or Alexa-Fluor 488-conjugated antibody (green), respectively. KANK1 was further visualized by anti-KANK1 antibody followed by Alexa-Fluor 555-conjugated antibody or Alexa-Fluor 488-conjugated antibody (shown in magenta). Finally, vinculin was visualized using conjugated anti-vinculin Alexa Fluor 647 antibody (shown in grey) and IRM images were taken. Analysis was performed using TCS SP8 Leica. Scale bar = 10 µm. (C) KANK1 knockdown does not affect sensitivity to PTX. Sensitivity of cells transfected with either control or KANK1-specific siRNA to PTX was measured by MTT assay. Twenty-four hours upon transfection, cells were seeded in 96-well plates and 24 h later treated with different concentrations of PTX. Data were analyzed by two-way analysis of variance (ANOVA) with Šídák’s multiple comparisons test, with a single pooled variance; ns, not significant; *P < 0.05; **P < 0.01; ***P < 0.001; ****P < 0.0001. Fig. S2. Cell area decreases upon knockdown of talin1, but not talin2. Quantification of data presented in Fig. 2. Violin plot represents measurements of > 30 cells (n = 2). Data were analyzed by one-way ANOVA with Dunnett’s multiple comparison. ns, not significant; *P < 0.05; **P < 0.01; ***P < 0.001; ****P < 0.0001. Fig. S3. The CMSC protein liprin-β1 loses its organization upon KANK2, but not upon talin2 knockdown. (A, B) Forty-eight hours after transfection with talin2 or KANK2-specific siRNA, MDA-MB-435S cells were fixed with methanol and stained with anti-liprin-β1 antibody followed by Alexa-Flour 546-conjugated antibody, anti-talin2 antibody followed by Alex [file 11658_2023_473_MOESM1_ESM.docx]

**Additional file 1**

**Talin2 and KANK2 functionally interact to regulate microtubule dynamics, paclitaxel sensitivity and cell migration in the MDA-MB-435S melanoma cell line**

Marija Lončarić^1, ORCID: 0000-0002-5343-0368^, Nikolina Stojanović^1, ORCID: 0000-0002-7763-4154^, Anja Rac-Justament^1, ORCID:0000-0001-8821-3059^, Kaatje Coopmans^1, ORCID: 0000-0002-8149-7818^, Dragomira Majhen^1, ORCID: 0000-0003-0385-0900^, Jonathan D. Humphries^2, ORCID:^ ^0000-0002-8953-7079^, Martin J. Humphries^3, ORCID: 0000-0002-4331-6967^, Andreja Ambriović-Ristov^1. #, ORCID: 0000-0001-7784-2466^

^1^Laboratory for Cell Biology and Signalling, Division of Molecular Biology, Ruđer Bošković Institute, Zagreb, Croatia; ^2^Department of Life Science, Manchester Metropolitan University, Manchester, United Kingdom; ^3^Wellcome Centre for Cell-Matrix Research, Faculty of Biology, Medicine & Health, University of Manchester, Manchester, United Kingdom

^#^corresponding author, [Andreja.Ambriovic.Ristov@irb.hr](mailto:Andreja.Ambriovic.Ristov@irb.hr)

**Supplementary Figures**

**
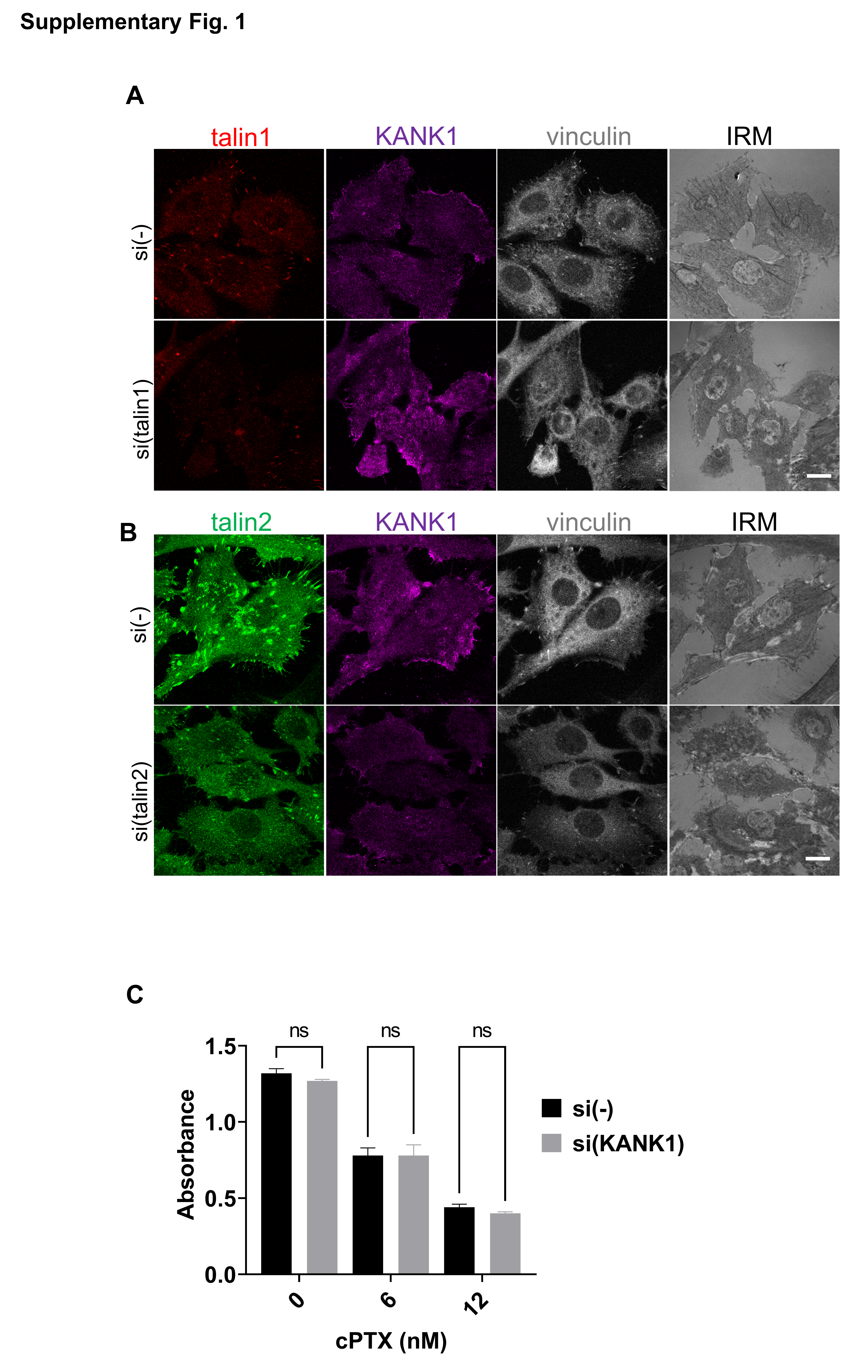
**

**Fig. S1** KANK1 localization is not affected by talin1 or talin2 knockdown. (A, B) Talin2, but not talin1, knockdown slightly affects KANK1 appearance. Forty-eight hours after transfection with either control, talin1 or talin2-specific siRNA, MDA-MB-435S cells were methanol fixed and stained with anti-talin1 or anti-talin2 antibody followed by Alexa-Fluor 546-conjugated antibody (red) or Alexa-Fluor 488-conjugated antibody (green), respectively. KANK1 was further visualized by anti-KANK1 antibody followed by Alexa-Fluor 555-conjugated antibody or Alexa-Fluor 488-conjugated antibody (shown in magenta). Finally, vinculin was visualized using conjugated anti-vinculin Alexa Fluor 647 antibody (shown in grey) and IRM images were taken. Analysis was performed using TCS SP8 Leica. Scale bar = 10 µm. (C) KANK1 knockdown does not affect sensitivity to PTX. Sensitivity of cells transfected with either control or KANK1-specific siRNA to PTX was measured by MTT assay. Twenty-four hours upon transfection, cells were seeded in 96-well plates and 24 h later treated with different concentrations of PTX. Data were analyzed by two-way analysis of variance (ANOVA) with Šídák’s multiple comparisons test, with a single pooled variance; ns, not significant; *P < 0.05; **P < 0.01; ***P < 0.001; ****P<0.0001.

**
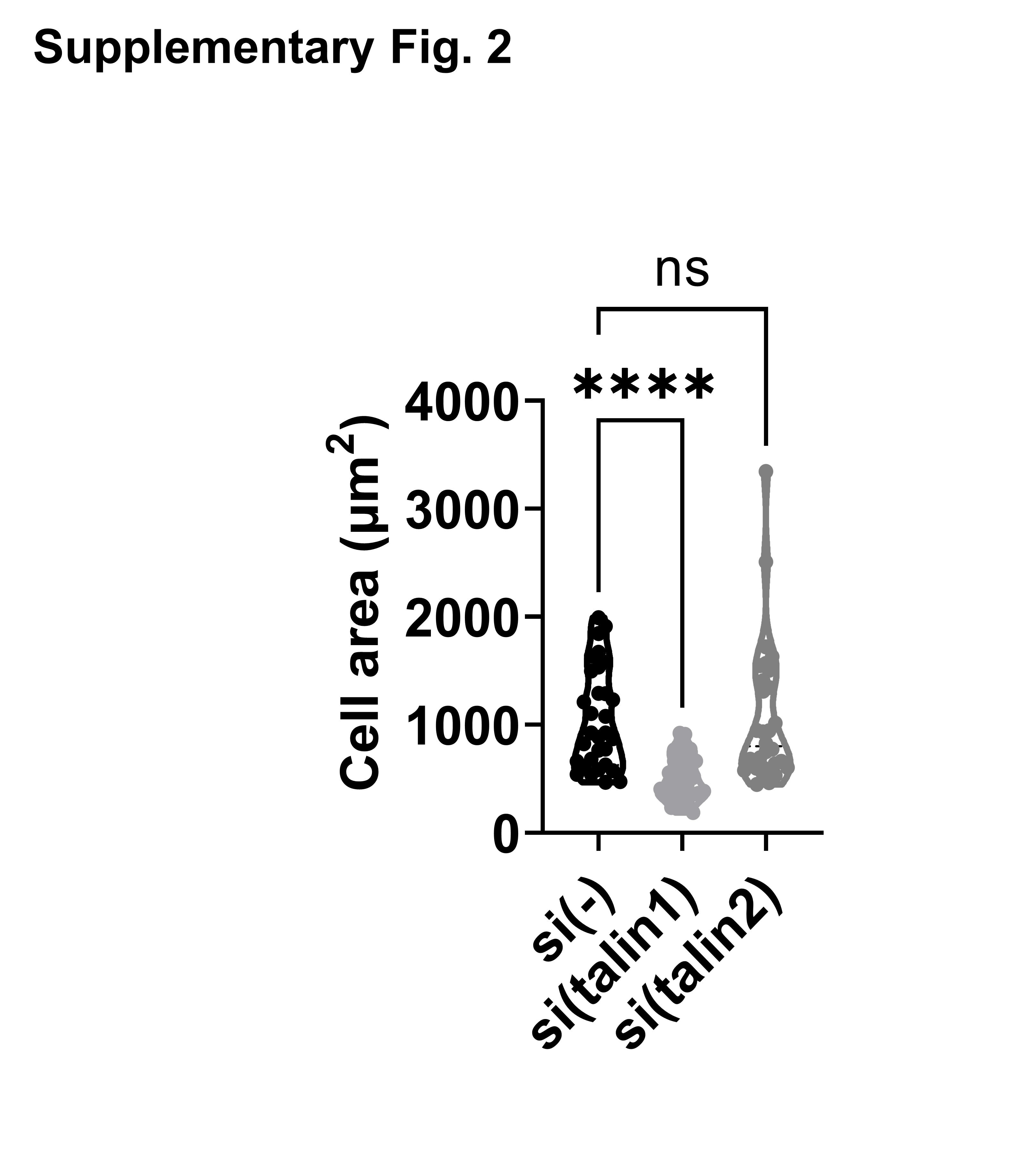
**

**Fig. S2** Cell area decreases upon knockdown of talin1, but not talin2. Quantification of data presented in Fig. 2. Violin plot represents measurements of > 30 cells (n = 2). Data were analyzed by one-way ANOVA with Dunnett’s multiple comparison. ns, not significant; *P < 0.05; **P < 0.01; ***P < 0.001; ****P<0.0001.

**
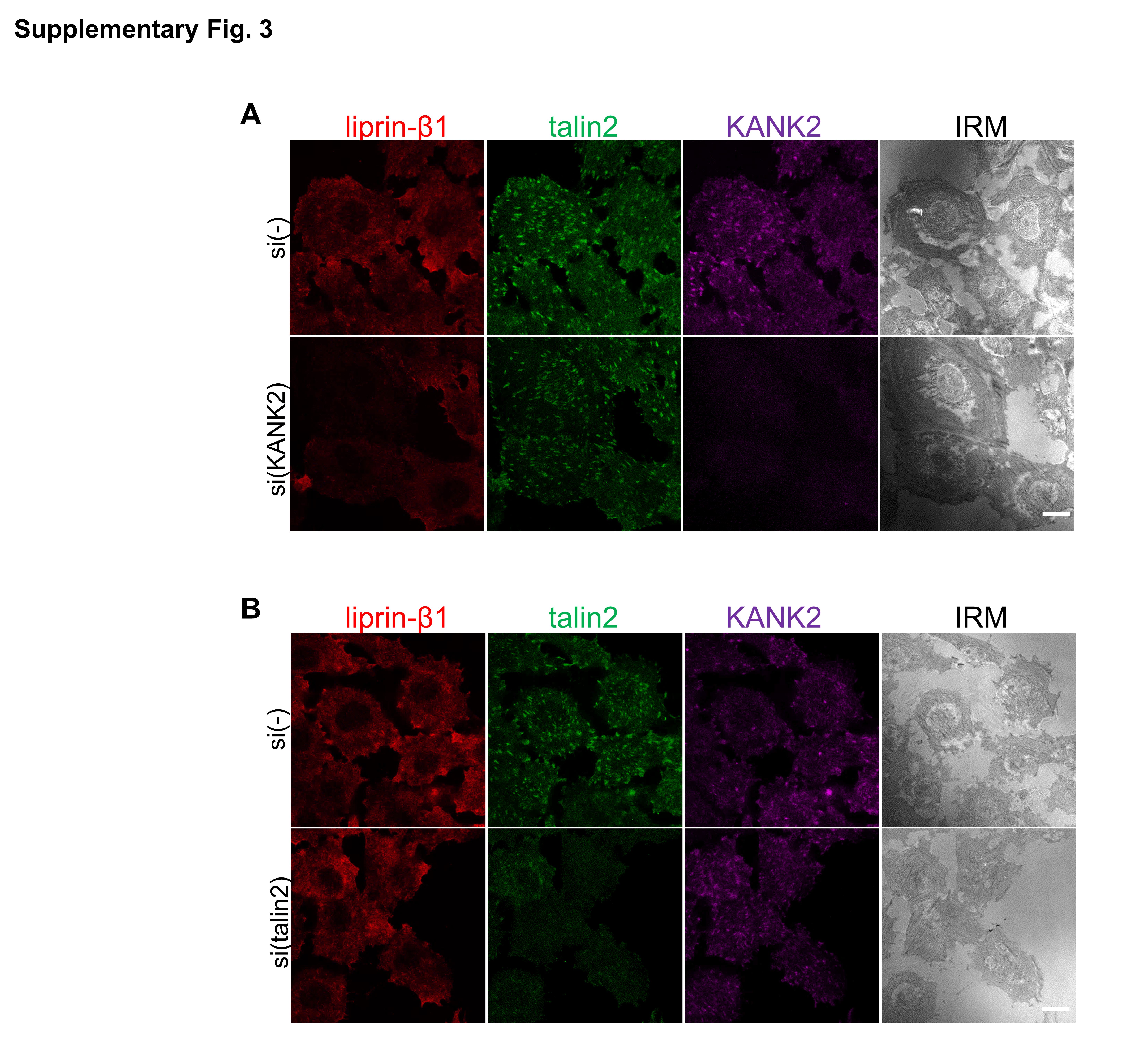
**

**Fig. S3** The CMSC protein liprin-β1 loses its organization upon KANK2, but not upon talin2 knockdown. (A, B) Forty-eight hours after transfection with talin2 or KANK2-specific siRNA, MDA-MB-435S cells were fixed with methanol and stained with anti-liprin-β1 antibody followed by Alexa-Flour 546-conjugated antibody, anti-talin2 antibody followed by Alexa-Fluor IgG2b 488-conjugated antibody (green) and anti-KANK2 antibody followed by Alexa-Flour 647-conjugated antibody and IRM images were taken. Analysis was performed using TCS SP8 Leica. Scale bar = 10 µm.

**
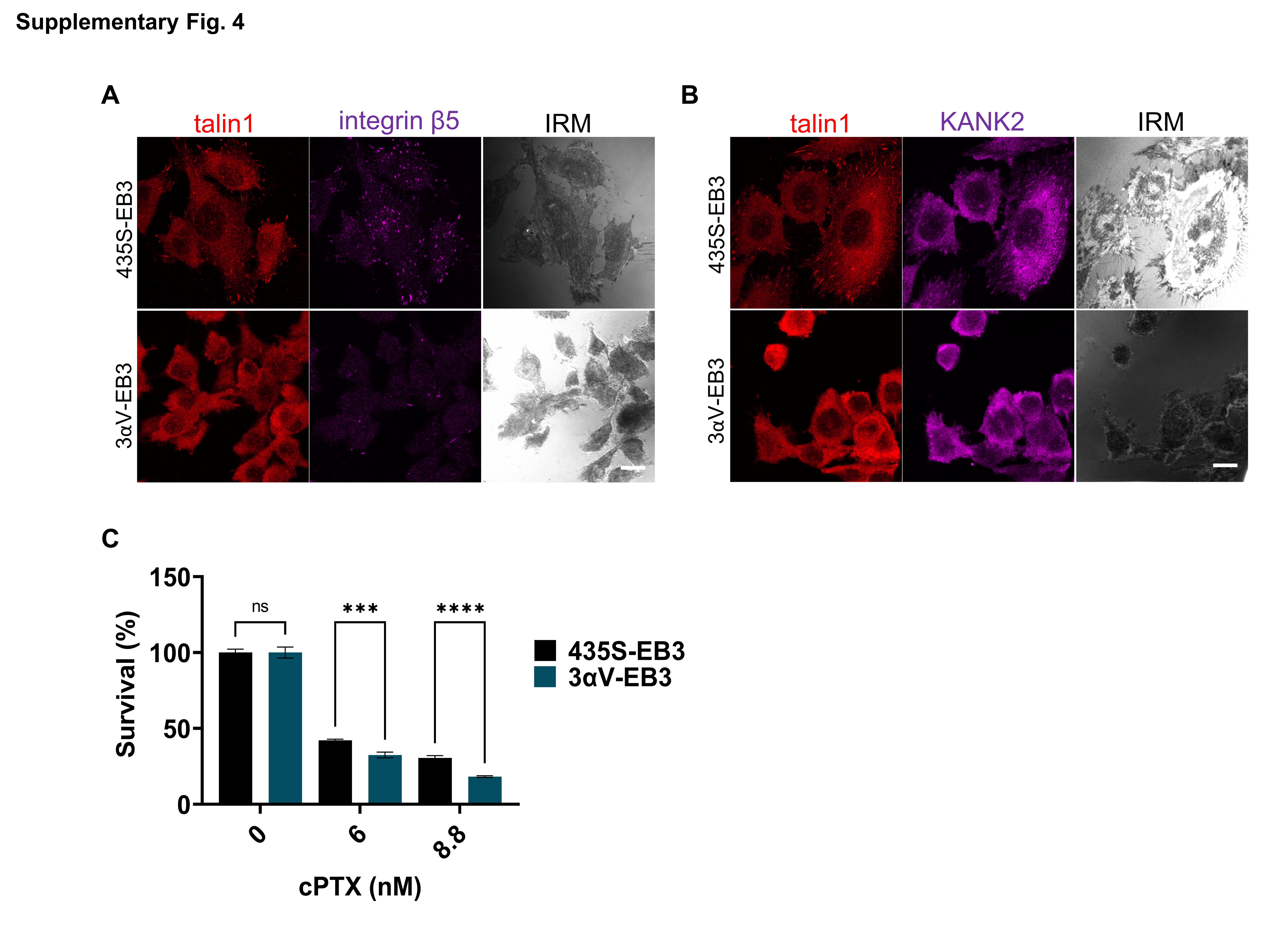
**

**Fig. S4** Verification of the MDA-MB-435S and 3αV cell model expressing fluorescently labelled EB3. (A, B) Clone 3αV-EB3 shows decreased expression of talin1, integrin β5 and KANK2 as compared to MDA-MB-435S-EB3 cells. Forty-eight hours after seeding cells were methanol fixed and stained with anti-talin1 followed by Alexa-Fluor 546-conjugated antibody (red), anti-KANK2 antibody or anti-β5 antibody followed by Alexa-Fluor 647-conjugated antibody (magenta) and IRM images were taken. Analysis was performed using TCS SP8 Leica. Scale bar = 10 µm. (C) Clone 3αV-EB3 demonstrates increased sensitivity to PTX as compared to parental MDA-MB-435S-EB3 cells. Twenty-four hours upon seeding in 96-well plates cells were treated with different concentrations of PTX. Cytotoxicity was measured by MTT assay. Data were analyzed by two-way analysis of variance (ANOVA) with Šídák’s multiple comparisons test, with a single pooled variance; ns denotes not significant; * P < 0.05; ** P < 0.01; *** P < 0.001; **** P < 0.0001 (n=3).

**
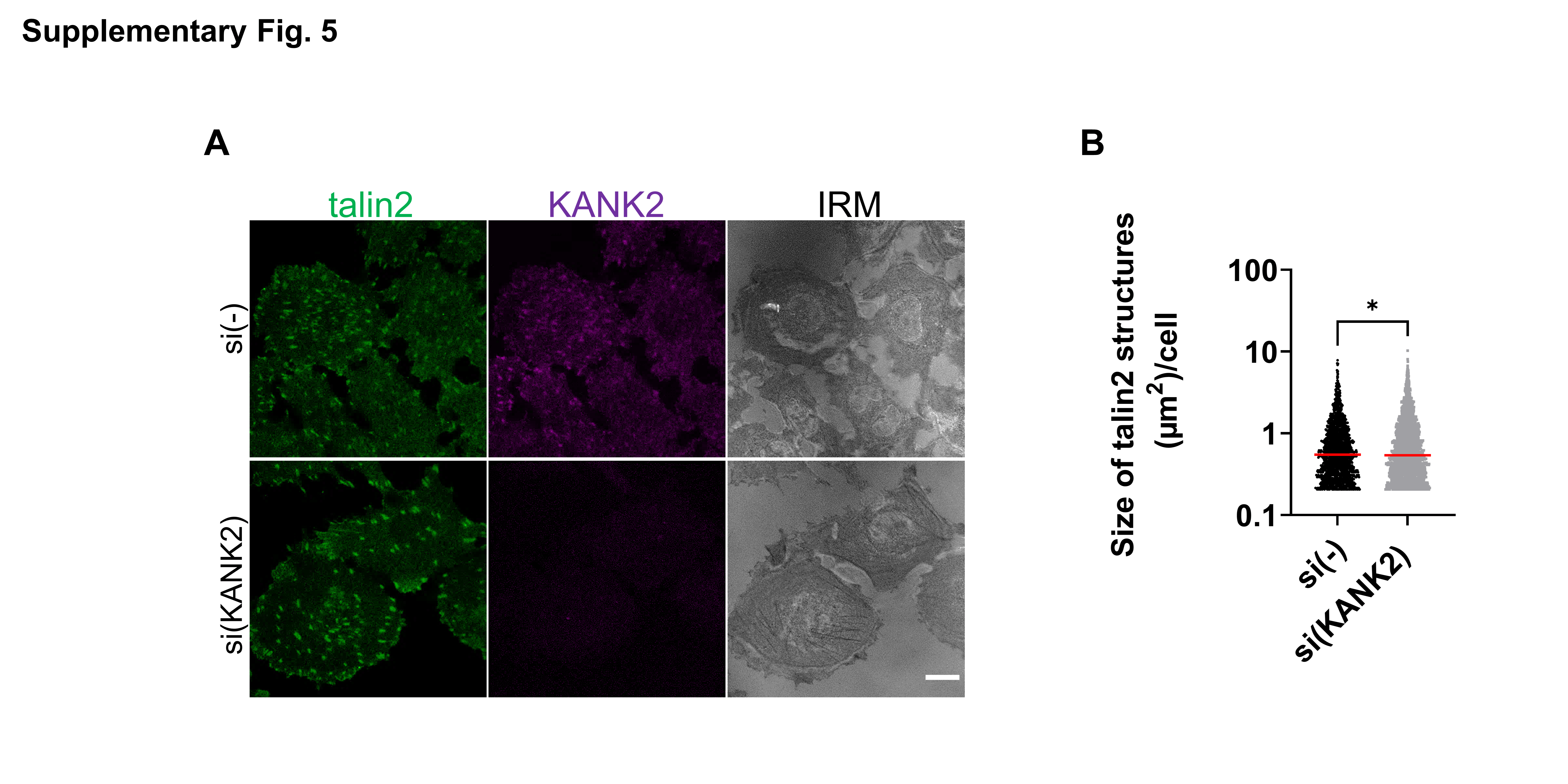
**

**Fig. S5** Knockdown of KANK2 increases talin2-positive FAs size. (A) Forty-eight hours after transfection with either control or KANK2-specific siRNA, MDA-MB-435S cells were methanol fixed and stained with anti-talin2 antibody followed by Alexa-Fluor IgG2b 488-conjugated antibody (green) and anti-KANK2 antibody followed by Alexa-Fluor 647-conjugated antibody (magenta) IRM images were taken. (B) Quantification of data presented in (A). Scatter plot with median marked in red represents measurements of ≥ 30 cells (n = 2). Data were analyzed by unpaired Student’s t-test. ns, not significant; *P < 0.05; **P < 0.01; ***P < 0.001; ****P<0.0001.





**Fig. S6** Full images of the blots in Fig. 2A. Images were obtained using Uvitec Alliance Q9 mini, which directly scanned membranes developed with ECL reagents.

**
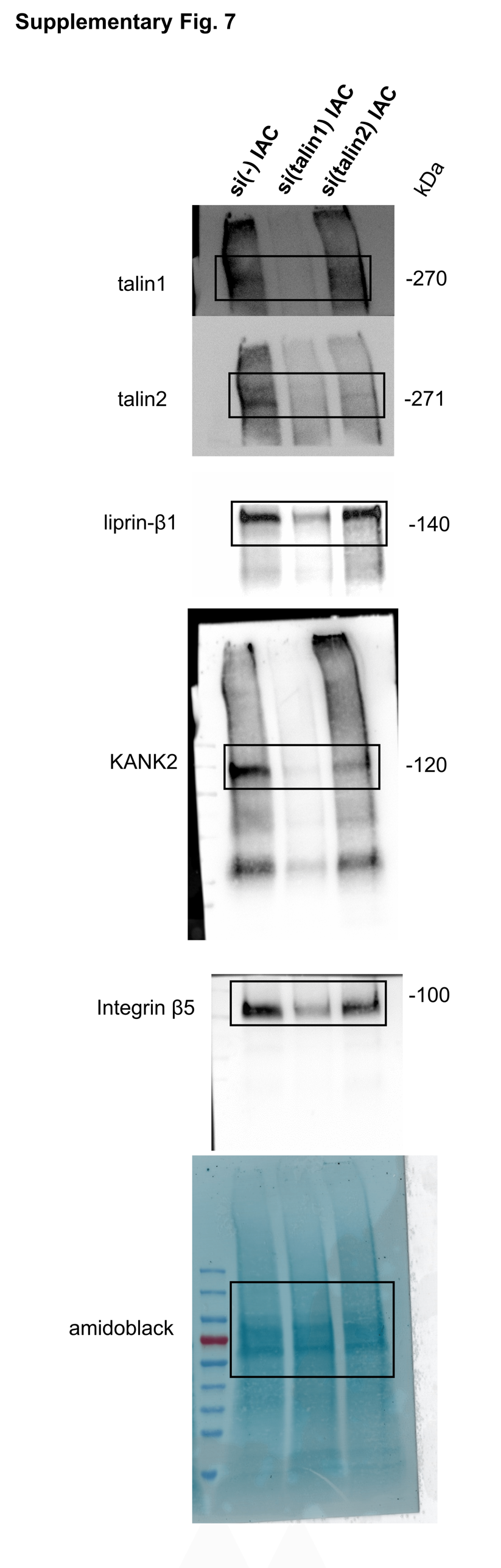
**

**Fig. S7** Full images of the blots in Fig. 3C. Images were obtained using Uvitec Alliance Q9 mini, which directly scanned membranes developed with ECL reagents.
