# Supplementary material for: Talin2 and KANK2 functionally interact to regulate microtubule dynamics, paclitaxel sensitivity and cell migration in the MDA-MB-435S melanoma cell line
Source: Cell Mol Biol Lett. 2023 Jul 17;28:56. doi: 10.1186/s11658-023-00473-6 (PMC10353188; doi:10.1186/s11658-023-00473-6)
Supplement: Supplementary file 8 — Additional file 8: Fig. S8. Still images of Additional file 2–Additional file 8: Movie S1–S6. Images were obtained using Image J manual tracking tool. Each arrow represent position of one microtubule tip through 104 s. Images were captured every 26 s. [file 11658_2023_473_MOESM8_ESM.docx]

**Additional file 8**

**Talin2 and KANK2 functionally interact to regulate microtubule dynamics, paclitaxel sensitivity and cell migration in the MDA-MB-435S melanoma cell line**

**Cellular & Molecular Biology Letters**

Marija Lončarić^1, ORCID: 0000-0002-5343-0368^, Nikolina Stojanović^1, ORCID: 0000-0002-7763-4154^, Anja Rac-Justament^1, ORCID:0000-0001-8821-3059^, Kaatje Coopmans^1, ORCID: 0000-0002-8149-7818^, Dragomira Majhen^1, ORCID: 0000-0003-0385-0900^, Jonathan D. Humphries^2, ORCID:^ ^0000-0002-8953-7079^, Martin J. Humphries^3, ORCID: 0000-0002-4331-6967^, Andreja Ambriović-Ristov^1. #, ORCID: 0000-0001-7784-2466^

^1^Laboratory for Cell Biology and Signalling, Division of Molecular Biology, Ruđer Bošković Institute, Zagreb, Croatia; ^2^Department of Life Science, Manchester Metropolitan University, Manchester, United Kingdom; ^3^Wellcome Centre for Cell-Matrix Research, Faculty of Biology, Medicine & Health, University of Manchester, Manchester, United Kingdom

^#^corresponding author, [Andreja.Ambriovic.Ristov@irb.hr](mailto:Andreja.Ambriovic.Ristov@irb.hr)

**Fig. S8** Still images of Additional File 2-8: Movie S1-S6. Images were obtained using Image J manual tracking tool. Each arrow represent position of one microtubule tip through 104 seconds. Images were captured every 26 seconds.
