# Supplementary material for: Talin2 and KANK2 functionally interact to regulate microtubule dynamics, paclitaxel sensitivity and cell migration in the MDA-MB-435S melanoma cell line
Source: Cell Mol Biol Lett. 2023 Jul 17;28:56. doi: 10.1186/s11658-023-00473-6 (PMC10353188; doi:10.1186/s11658-023-00473-6)
Supplement: Supplementary file 9 — Additional file 9: Table S1. List of used antibodies and dyes. [file 11658_2023_473_MOESM9_ESM.docx]

**Additional file 9**

**Talin2 and KANK2 functionally interact to regulate microtubule dynamics, paclitaxel sensitivity and cell migration in the MDA-MB-435S melanoma cell line**

**Cellular & Molecular Biology Letters**

Marija Lončarić^1, ORCID: 0000-0002-5343-0368^, Nikolina Stojanović^1, ORCID: 0000-0002-7763-4154^, Anja Rac-Justament^1, ORCID:0000-0001-8821-3059^, Kaatje Coopmans^1, ORCID: 0000-0002-8149-7818^, Dragomira Majhen^1, ORCID: 0000-0003-0385-0900^, Jonathan D. Humphries^2, ORCID:^ ^0000-0002-8953-7079^, Martin J. Humphries^3, ORCID: 0000-0002-4331-6967^, Andreja Ambriović-Ristov^1. #, ORCID: 0000-0001-7784-2466^

^1^Laboratory for Cell Biology and Signalling, Division of Molecular Biology, Ruđer Bošković Institute, Zagreb, Croatia; ^2^Department of Life Science, Manchester Metropolitan University, Manchester, United Kingdom; ^3^Wellcome Centre for Cell-Matrix Research, Faculty of Biology, Medicine & Health, University of Manchester, Manchester, United Kingdom

^#^corresponding author, [Andreja.Ambriovic.Ristov@irb.hr](mailto:Andreja.Ambriovic.Ristov@irb.hr)

**Supplementary Table**

**Table S1.**  List of used antibodies and dyes.

| **WESTERN BLOT** | | | | | |
| --- | --- | --- | --- | --- | --- |
| ***Primary antibodies*** | ***Ref. No.*** | ***Distributor*** | ***Monoclonal/***  ***polyclonal*** | ***Species*** | ***Dilution*** |
| Anti-KANK1 | HPA005539 | Sigma-Aldrich, USA | Polyclonal | Rabbit | 1:450 in 5% milk |
| Anti-KANK2 | HPA015643 | Sigma-Aldrich, USA | Polyclonal | Rabbit | 1:1000 in 5% milk |
| Anti-human talin1 | MCA4770GA | Bio-Rad, USA | Monoclonal | Mouse | 1:1000 in 5% milk |
| Anti-human talin2 | MCA4771GA | Bio-Rad, USA | Monoclonal | Mouse | 1:1000 in 5% milk |
| Integrin β5 | D24A5 | Cell Signaling Technology, USA | Monoclonal | Mouse | 1:1000 in 5% milk |
| Anti-Liprin β1 | sc-514575 | Santa Cruz Biotechnology, USA | Monoclonal | Mouse | 1:100 in 5% milk |
| ***Secondary antibodies*** | ***Ref. No.*** | ***Distributor*** | ***Monoclonal/***  ***polyclonal*** | ***Species*** | ***Dilution*** |
| Goat anti-rabbit IgG (H+L) | 31466 | Invitrogen, USA | Polyclonal | Goat | 1:5000 in 5% milk |
| Goat anti-mouse IgG (H+L) | G21040 | Invitrogen, USA | Polyclonal | Goat | 1:10 000 in 5% milk |
| **IMMUNOFLUORESCENCE** | | | | | |
| ***Primary antibodies*** | ***Ref. No.*** | ***Distributor*** | ***Monoclonal/***  ***polyclonal*** | ***Species*** | ***Dilution*** |
| Anti-KANK1 | HPA005539 | Sigma-Aldrich, USA | Polyclonal | Rabbit | 1:100 in 5% BSA |
| Anti-KANK2 | HPA015643 | Sigma-Aldrich, USA | Polyclonal | Rabbit | 1:100 in 5% BSA |
| Anti-human talin1 | MCA4770GA | Bio-Rad, USA | Monoclonal | Mouse | 1:100 in 5% BSA |
| Anti-human talin2 | MCA4771GA | Bio-Rad, USA | Monoclonal | Mouse | 1:100 in 5% BSA |
| Integrin β5 | D24A5 | Cell Signaling Technology, USA | Monoclonal | Rabbit | 1:800 in 5% BSA |
| Anti-Liprin β1 | sc-514575 | Santa Cruz Biotechnology, USA | Monoclonal | Mouse | 1:50 in 5% BSA |
| Anti-alpha tubulin | ab52866 | Abcam, UK | Polyclonal | Rabbit | 1:250 in 5% BSA |
| Anti-alpha tubulin | CP06 | Sigma-Aldrich, USA | Monoclonal | Mouse | 1:20 in 5% BSA |
| Recombinant Alexa Fluor® 647 Anti-Vinculin | ab196579 | Abcam, UK | Monoclonal | Rabbit | 1:200 in 5% BSA |
| Recombinant Alexa Fluor® 555 Anti-Vinculin | ab206908 | Abcam, UK | Monoclonal | Rabbit | 1:200 in 5% BSA |
| ***Secondary antibodies*** | ***Ref. No.*** | ***Distributor*** | ***Monoclonal/***  ***polyclonal*** | ***Species*** | ***Dilution*** |
| Anti-Mouse IgG Alexa Fluor 546 | A-11030 | Invitrogen, USA | Polyclonal | Goat | 1:1000 in 5% BSA |
| Anti-Mouse IgG Alexa Fluor 488, | #4408 | Cell Signaling Technology, USA |  | Goat | 1:1000 in 5% BSA |
| Anti-Mouse IgG Alexa Fluor 405 | A-31553 | Invitrogen, USA | Polyclonal | Goat | 1:250 in 5% BSA |
| Anti-Rabbit IgG Alexa Flour, 555 | A-31572 | Invitrogen, USA | Polyclonal | Donkey | 1:1000 in 5% BSA |
| Anti-Rabbit IgG Alexa Fluor 647 | #4414 | Cell Signaling Technology, USA | Polyclonal | Goat | 1:1000 in 5% BSA |
| Anti-Mouse IgG1 Alexa Fluor 555, | A-21127 | Invitrogen, USA | Polyclonal | Goat | 1:1000 in 5% BSA |
| Anti-Mouse IgG2_b_ Alexa Fluor 488 | A-21141 | Invitrogen, USA | Polyclonal | Goat | 1:1000 in 5% BSA |
| ***Dyes*** | ***Ref. No.*** | ***Distributor*** |  |  | ***Dilution*** |
| Phalloidin, Alexa Fluor 488 | P5282 | Sigma Aldrich, USA |  |  | 1:100 in 5% BSA |
